# Plant mitochondrial introns as genetic markers - conservation and variation

**DOI:** 10.3389/fpls.2023.1116851

**Published:** 2023-03-20

**Authors:** Melinda R. Grosser, Samantha K. Sites, Mayara M. Murata, Yolanda Lopez, Karen C. Chamusco, Kyra Love Harriage, Jude W. Grosser, James H. Graham, Fred G. Gmitter, Christine D. Chase

**Affiliations:** ^1^ Horticultural Sciences Department, University of Florida, Gainesville, FL, United States; ^2^ Citrus Research and Education Center, University of Florida, Lake Alfred, FL, United States; ^3^ Agronomy Department, University of Florida, Gainesville, FL, United States

**Keywords:** group II intron, indel polymorphism, organelle genome, PCR-based markers, plant mitochondria, single nucleotide polymorphism

## Abstract

Plant genomes are comprised of nuclear, plastid and mitochondrial components characterized by different patterns of inheritance and evolution. Genetic markers from the three genomes provide complementary tools for investigations of inheritance, genetic relationships and phenotypic contributions. Plant mitochondrial genomes are challenging for universal marker development because they are highly variable in terms of size, gene order and intergenic sequences and highly conserved with respect to protein-coding sequences. PCR amplification of introns with primers that anneal to conserved, flanking exons is effective for the development of polymorphic nuclear genome markers. The potential for plant mitochondrial intron polymorphisms to distinguish between congeneric species or intraspecific varieties has not been systematically investigated and is possibly constrained by requirements for intron secondary structure and interactions with co-evolved organelle intron splicing factors. To explore the potential for broadly applicable plant mitochondrial intron markers, PCR primer sets based upon conserved sequences flanking 11 introns common to seven angiosperm species were tested across a range of plant orders. PCR-amplified introns were screened for indel polymorphisms among a group of cross-compatible *Citrus* species and relatives; two *Raphanus sativus* mitotypes; representatives of the two *Phaseolus vulgaris* gene pools; and congeneric pairs of *Cynodon*, *Cenchrus*, *Solanum*, and *Vaccinium* species. All introns were successfully amplified from each plant entry. Length polymorphisms distinguishable by gel electrophoresis were common among genera but infrequent within genera. Sequencing of three introns amplified from 16 entries identified additional short indel polymorphisms and nucleotide substitutions that separated *Citrus*, *Cynodon*, *Cenchrus* and *Vaccinium* congeners, but failed to distinguish *Solanum* congeners or representatives of the *Phaseolus vulgaris* major gene pools. The ability of primer sets to amplify a wider range of plant species’ introns and the presence of intron polymorphisms that distinguish congeners was confirmed by in silico analysis. While mitochondrial intron variation is limited in comparison to nuclear introns, these exon-based primer sets provide robust tools for the amplification of mitochondrial introns across a wide range of plant species wherein useful polymorphisms can be identified.

## Introduction

Plant genetic information is distributed among nuclear, plastid and mitochondrial genomes (Mahapatra et al., 2021; Camus et al., 2022), and genetic markers for each genome provide complementary tools for investigations of inheritance and evolution (Qiu et al., 1999; Duminil and Besnard, 2021; Besse, 2021; Camus et al., 2022). Although genome sequencing is the gold standard for such studies, convenient PCR-based markers retain utility and appeal (Egan et al., 2012; Hodel et al., 2016; Besse, 2021). The distinctive features of each genome necessitate different strategies for marker development and create different opportunities for application. Plant plastid genomes are relatively conserved in gene order, moderately conserved in coding sequences and more polymorphic with respect to introns and intergenic spacers (Wicke et al., 2011), facilitating the development of universal primers for the PCR amplification and subsequent characterization of more variable regions. Amplified plastid sequences such as *rbc*L, *mat*K are the core of the DNA barcoding approach for distinguishing plant species (CBOL Plant Working Group, 2009), with the BOLD database facilitating applications (Ratnasingham and Hebert, 2007), and with intergenic spacer regions proving more variable and useful in distinguishing closer relatives (Shaw et al., 2014). Barcoding is also being combined with plastid genome sequencing for broader applicability and enhanced resolution (Tonti-Filippini et al., 2017). Plant mitochondrial markers provide an important adjunct to plastid DNA markers (Duminil and Besnard, 2021). Mitochondrial genotype can have a significant influence on plant phenotype (Bock et al., 2014; Colombatti et al., 2014; Hu et al., 2014; Dourmap et al., 2020). It is therefore important to be able to track mitochondrial contributions in sexual crosses and somatic cell fusions. Both plastid and plant mitochondrial genomes have uni-parental inheritance patterns, but these are not always concordant with respect to parent of origin (Camus et al., 2022). Moreover, horizontal gene transfer, observed in both organelle genomes, is especially prevalent in plant mitochondria (Keeling, 2009; Archibald and Richards, 2010). Plant mitochondrial gene coding sequences are, with some exceptions (Mower et al., 2007), highly conserved (Wolfe et al., 1987; Drouin et al., 2008), but genome size, gene order and intergenic sequences vary exensively between, and even within, plant species (Sloan, 2013; Gualberto and Newton, 2017). Mitochondrial restriction fragment length polymorphisms (RFLPs) are therefore readily detected within plant species (Levings and Pring, 1977; Palmer and Herbon, 1988), but the development of polymorphic PCR-based mitochondrial markers that work across a wide range of plant species is problematic. The lack of conserved gene order precludes the development of universal primer sets that will anneal to conserved coding sequences and amplify the highly polymorphic intergenic sequences.

Minisatellites and microsatellites (tandem repeats of 10 to 100, or less than 10 base pairs, respectively) identified within the sequenced mitochondrial genomes of some plant species have provided the basis for PCR-based polymorphic markers. Minisatellite repeat number polymorphisms have demonstrated intraspecific variation in *Beta vulgaris*, *B. maritima* (Nishizawa et al., 2000; Nishizawa et al., 2007), *Picea abies* (Sperisen et al., 2001; Bastien et al., 2003), *Pinus banksiana* (Godbout et al., 2005), and *Pinus ponderosa* (Mitton et al., 2000), as well as interspecific polymorphisms in *Brassica* and *Oryza* species (Honma et al., 2011). Interspecific, but not intraspecific, variation for a G_n_ microsatellite is present in the genus *Pinus* (Soranzo et al., 1999), whereas a compound, highly polymorphic microsatellite region reveals both intra- and interspecific variation in *Abies* (Jaramillo-Correa et al., 2013). Tandemly repeat mitochondrial loci are not generally conserved across diverse plant taxa and are not always polymorphic between related taxa, but recent work has identified extensive mitochondrial microsatellites among plant species (de Freitas et al., 2022; Xiong et al., 2022). These studies and databases of plant mitochondrial microsatellite repeats (Kumar et al., 2014; Sablok et al., 2015) facilitate the experimental search for loci that are polymorphic in specific taxa.

Plant mitochondrial introns present an under-explored approach for the development of more universal, PCR-based mitochondrial genome markers. PCR amplification of polymorphic introns with primers designed to conserved flanking exon sequences (Lessa, 1992) has allowed the development of nuclear genome markers in plant species having limited genomic information (Gupta et al., 2011; Li et al., 2012; Chandra et al., 2013; Kim et al., 2015) or limited genetic variability (Wang et al., 2010; Galeano et al., 2012). Angiosperm mitochondrial genomes encode 20-24 group II introns. Although sporadic intron loss is observed among evolutionary lineages, many of these introns are common to the sequenced angiosperm mitochondrial genomes (Kubo and Mikami, 2007), and flanked by conserved coding sequences that can be exploited for universal primer development. Laroche et al. (1997) surveyed the genomic sequences of six mitochondrial introns that were located within five genes and were common to five different angiosperm species and concluded that plant mitochondrial introns could provide a source of polymorphic markers. Across these species, base substitutions per site were higher within introns than within exons. Insertion-deletion (indel) polymorphisms were observed at 0.2-0.5 times the frequency of base substitutions. These sequence comparisons were made across a small set of diverse angiosperm genera, and so did not determine whether plant mitochondrial introns are commonly polymorphic between cross-compatible species or within species – situations in which polymorphisms could function as useful genetic markers. These points require investigation as correct splicing of plant organelle group II introns depends upon a complex intron secondary structure and upon RNA-protein interactions with multiple, co-evolving, nuclear-encoded splicing factors (Bonen, 2008; de Longevialle et al., 2010; Brown et al., 2014) –requirements that potentially constrain the degree of intron polymorphism that can be found among close relatives.

DNA markers based upon PCR-amplified plant mitochondrial intron sequences have proved useful in some cases. While most plant mitochondrial microsatellite and minisatellite repeats are located in intergenic regions, polymorphic examples are found within introns (Sperisen et al., 2001; Godbout et al., 2005; Jaramillo-Correa et al., 2013; Potter et al., 2013; Xiong et al., 2022). Duminil et al. (2002) designed primer pairs for the amplification of 16 different introns, based upon the mitochondrial genome sequences of *Arabidopsis thaliana* and *Beta vulgaris*. These primer sets amplify their corresponding introns in 20-28 of 28 diverse angiosperm species, and some have been investigated for polymorphisms in related species. The PCR amplified NADH dehydrogenase subunit 1 intron 2 (*nad1*i2), NADH dehydrogenase subunit 4 intron 1 (*nad4*i1) and intron 2 (*nad4*i2) are not polymorphic within *Quercus robur*, but distinguish between *Q. robur* and *Q. rubra* (Demesure et al., 1995). Notably, complex mitochondrial SSR loci analyzed across 88 genomes are especially prevalent in the introns of *nad2*, *nad4* and *nad7* genes (Xiong et al., 2022).

Mitochondrial intron polymorphisms also have utility in citrus breeding and genetics. Commercial citrus types are complex hybrids with at least three maternal lineages among them - *Citrus maxima* (pummelo), *C. reticulata* (mandarin) and *C. medica* (citron). The genus overall has complex taxonomy (Moore, 2001; Wu et al., 2018). Froelicher et al. (2011) amplified short, internal, regions of *Citrus* NADH dehydrogenase subunit 2 intron 3 (*nad2*i3), NADH dehydrogenase subunit 5 intron 2 (*nad5*i2), and NADH dehydrogenase subunit 7 intron 1 (*nad7*i1), with primers based upon *A. thaliana* and *B. vulgaris* mitochondrial genome sequences. *Citrus* and citrus relatives are polymorphic for indels in these introns, which collectively identify seven *Citrus* mitotypes. Intron-flanking primers designed from alignment of conserved DNA sequences flanking introns common to seven sequenced angiosperm mitochondrial genomes (Grosser, 2011) generate intron amplification products that distinguish *C. maxima* from *C. reticulata* (Satpute et al., 2015) and *C. maxima* from *C. japonica* (kumquat) (Omar et al., 2017). Here, we demonstrate the utility of these primer sets for amplification of their target introns not only in the previously studied *C. maxima*, *C. reticulata* and *C. Japonica* lineages, but also across diverse angiosperm species. We further investigate the amplified introns for indel and single nucleotide polymorphisms (SNPs) that distinguish mitochondrial genomes within a plant species or between congeneric plant relatives, wherein polymorphic mitochondrial markers have potential applications in studies of evolution and inheritance.

## Materials and methods

### Plant materials and DNA extraction

The plant materials used in this study (**Table 1**) were selected to explore primer amplification across across six angiosperm orders and to investigate whether intron amplification products could, at least, distinguish congener species of agricultural importance within these orders. These included two commercial *Raphanus sativus* mitotypes confirmed by PCR markers as described by Kim et al. (2007), representatives of the two major *Phaseolus vulgaris* gene pools (Bhakta et al., 2017), congener species representatives of *Cenchrus, Citrus*, *Cynodon*, *Solanum* and *Vaccinium*, along with *Poncirus trifoliata*, which is cross-compatible with *Citrus* species (Moreira et al., 2002) and considered by some to fall within the genus *Citrus* (Ollitrault et al., 2020). *Citrus* materials were from the University of Florida Citrus Research and Education Center, Lake Alfred, Florida and Harris Citrus Nursery, Lithia, FL. The *Cynodon* entries were from the USDA National Plant Germplasm System. The *Phaseolus*, *Cenchrus*, *Solanum* and *Vaccinium* entries were obtained from the University of Florida research programs of Dr. C.E. Vallejos, Dr. L. Sollenberger, Dr. C.E. Vallejos, and Dr. J. Olmstead, respectively. Total cellular DNA was extracted from leaf samples by a modification of the cetyl trimethylammonium bromide (CTAB) method in which 50 mg of tissue was combined with 750 μl of CTAB buffer (Murray et al., 1980) and 10 μg of DNase-free RNase A in a FastPrep™ Lysing Matrix A tube, disrupted for 40 s in a FastPrep^®^-24 Instrument (MP Biomedicals LLC, Santa Ana, CA) and incubated at 65°C for 5 min. Cellular and lysing matrix debris was removed by centrifugation at 13,000 xg for 10 min at room temperature. Supernatants were extracted with an equal volume of chloroform-isoamyl alcohol mixed in a ratio of 24:1. DNA was precipitated from the aqueous phase by the addition of a 2/3 volume of isopropyl alcohol and recovered by centrifugation at 13,000 xg for 10 min at room temperature. The pellets were washed in 750 μl of 70% ethanol, air dried and rehydrated in 80 ul of 1 mM Trizma base, 0.1 mM di-sodium ethylene diamine tetra acetic acid (Na_2_EDTA), 1 mM NaCl, pH 8. The concentration of DNA samples was determined from the absorbance at 260 nm.

**Table 1:** Plant materials and intron sequence GenBank accession numbers.

Genus Species	Cultivar/Accession	GenBank Accession		
		*ccmFc*i1	*nad5*i4	*nad7*i1
*Cenchrus amercianus* ^a^	TifLeaf3	OP800670	OP800688	OP800704
*Cenchrus purpureus*	Merkeron	OP800671	OP800689	OP800705
*Citrus maxima*	Hirado Buntan Pummelo	OP800658	OP800674	OP800690
*Citrus japonica*	Meiwa	OP800662	OP800678	OP800694
*Citrus medica*	Etrog	OP800661	OP800677	OP800693
*Citrus paradisi* ^b^	Ruby Red	OP800659	OP800675	OP800691
*Citrus reticulata*	Ponkan	OP800660	OP800676	OP800692
*Citrus sinensis* ^c^	Valencia	ND^d^	ND	ND
*Cynodon dactylon*	Royal Cape/PI290868	OP800668	OP800686	OP800702
*Cynodon transvaalensis*	Frankenwald Fine/PI290905	OP800669	OP800687	OP800703
*Phaseolus vulgaris*	Jamapa (Mesoamerican)	OP800673	OP800685	OP800701
*Phaseolus vulgaris*	Calima (Andean)	OP800672	OP800684	OP800700
*Poncirus trifoliata* ^e^	English Large Flower	OP800663	OP800679	OP800695
*Raphanus sativus*	Red Velvet^f^	ND	ND	ND
*Raphanus sativus*	April Cross^g^	ND	ND	ND
*Solanum lycopersicum*	Bonny Best	OP800664	OP800680	OP800696
*Solanum pennellii*	LA716	OP800665	OP800681	OP800697
*Vaccinium corymbosum*	Bluecrop	OP800666	OP800682	OP800698
*Vaccinium virgatum*	Tifblue	OP800667	OP800683	OP800699

a *Cenchrus americanus* (*Pennisetum glaucum*, pearl millet) hybrid with wild *P. americanum* subsp. *Monodii* cytoplasm (Hanna, 1997; Hanna et al., 1997).

b *Citrus maxima* maternal lineage.

c *Citrus reticulata* maternal lineage.

d ND, sequence not determined.

e Considered by some as *Citrus trifoliata* (Ollitrault et al., 2020).

f F1 hybrid Harris Seeds 11701-00-00; commercial seed mixture or heteroplasmy prevented acquiring intron sequences.

g F1 Hybrid Harris Seeds 11700-00-01: commercial seed mixture or heteroplasmy prevented acquiring intron sequences.

### DNA amplification and fractionation

The PCR primers used in this work (**Table 2**; Grosser, 2011) were designed against introns of the mitochondrial *nad1, nad2, nad4, nad5, nad7* and *cyctochrome c maturation Fc* (*ccmFc*) genes because these introns were common to seven plant species’ mitochondrial genomes: *A. thaliana* (Unseld et al., 1997), *B. napus* (Handa, 2003)*, B. vulgaris* (Kubo et al., 2000)*, N. tabacum* (Sugiyama et al., 2005)*, O. sativa* (Notsu et al., 2002)*, T. aestivum* (Ogihara et al., 2005), and *Z. mays* (Allen et al., 2007). The National Center for Biotechnology Information (NCBI) accession numbers for these genomes are NC_001284, NC_002511, NC_008285, NC_006581, NC_007886, NC_007579, and NC_007982, respectively (https://www.ncbi.nlm.nih.gov/genome/organelle/, accessed 1/20/2023). Primer pairs were designed manually based upon the highly conserved coding regions flanking intron sequences or, in some cases, from conserved sequences within introns.

**Table 2:** Primers for amplification and sequencing of plant mitochondrial introns.

Intron	Forward primer sequence (5’-3’)	Reverse primer sequence (5’-3’)
*ccmFc*i1	TTTCACATGGAGGAGTGTGC	TTCCCCATATGGAGTTCG
*ccmFc*i1	ATTGGTCAGACGACGACTACT^a^	TCTCTCAGTGTGGTCAGC^a^
*nad1*i2	CGATCTGCAGCTCAAATGGT	ACCTACAGCCCTTTCCTCT
*nad2*i1	GTAATGTGGGTTGGCTTGGA	GCAATAGTTAGGAGAGGTG
*nad2*i4	CAGTGGGAGTAGTGACTAG	GGAAGTCATTGCTAGTAG
*nad4*i1	AGGGGCCTTGTGCAGTAAA^b^	CTTTCTTTGTCTCGAACCCC
*nad4*i3	GTAGTACCGGTGAACCAGAT^b^	CTTACGGATGTATGCATG
*nad5*i1	ATGTTTGATGCTTCTTGGGG	TTAACATCACTACGGTCGGG
*nad5*i4	GGTATCTCGTACACATTCCG	CCCACATACGAGAAAAGGTC
*nad5*i4	CAACTAGTATAGTATAGCAG^a^	GGGAATCTAGGAATGAATGG^a^
*nad7*i1	AACGGAGAAGTGGTGGAACG	TTTCTCAGTCCCTCTAGTCG
*nad7*i1	AAGACCGTCTGGCGAAAACG^a^	CGTTTTCGCCAGACGGTCTT^a^
*nad7*i2	AGATGCCAGCGGAATGAT	GTGTTCTTGGGCCATCATAG
*nad7*i3	ATGTTAAGAGGTCGTGCG	AACATCGTAAGGTGCTGCTC

a Internal primer for intron sequencing.

b Primer binds near terminus but within the target intron.

PCR amplification reactions were performed on replicate DNA preparations made from different plants of each entry, with the exception of the two *Cynodon* entries. For these only a single pot culture was available, so replicate DNA extractions were prepared from the single culture of each. PCR reactions of 50 μl contained 25-100 ng of DNA, 0.2 µM of each primer, 0.125 mM dNTPs, 1.25 units of high fidelity, TAKARA EXTAQ Hot Start DNA polymerase (Clontech, Mountain View, CA) in 1X TAKARA Hot Start reaction buffer. This high-fidelity polymerase was selected due to the length of the amplified introns and the intent to sequence PCR products. Amplification was for 30 cycles of 1 min at 94°C, 2 min at 55°C, and 3 min at 72°C. Electrophoresis through 1% agarose gels was performed to survey PCR reactions for successful amplification. The DNA Hyperladder II (Bioline Inc., Cambridge, MA) was used as a size marker. Electrophoresis was at 100V for 100 min in Tris-Borate-EDTA (TBE) buffer (10 mM Trizma base, 10 mM boric acid, 2.5 mM Na_2_EDTA, pH 8.2). Gels were stained in 0.5 μg/ml ethidium bromide for 20 min and viewed over a UV transilluminator in a Molecular Imager^®^ Gel Doc™ XR System (Bio Rad Laboratories, Inc. Hercules, CA). Gel images were captured with the Quantity One^®^ 1-D Analysis Software (Bio Rad Laboratories, Inc.) and exported as.tif files. The AdvanCE™ FS96 capillary electrophoresis system (Advanced Analytical Technologies Inc., Ames, IA) was used to estimate the length of PCR amplification products in DNA base pairs (bp). Amplification products were diluted 1:15 in TE buffer (10 mM Trizma Base 1 mM Na_2_EDTA, pH 8) and fractionated by use of the DNF-915 dsDNA 915 Reagent Kit (Advanced Analytical Technologies Inc.) according to the supplier’s instructions. Indel polymorphisms were confirmed by electrophoresis of DNA amplification products, individually and mixed, through Criterion™ precast 5% polyacrylamide gels (Bio-Rad Laboratories Inc., Hercules, CA) run in TBE buffer for 740 Volt-h and imaged as described above.

### DNA sequencing and sequence analysis

The amplification products of *ccmFc*i1, *nad5*i4, *nad7*i1were purified for DNA sequencing through use of the QIAquick PCR Purification Kit (Qiagen Inc., Valencia, CA) according to supplier’s instructions. Purified amplification products were fully sequenced in both directions by the University of Florida Interdisciplinary Center for Biotechnology Research (ICBR) Sanger Sequencing Core Laboratory in Gainesville, FL or by Eurofins USA. Intron sequences and their corresponding GenBank Accession numbers are listed in **Table 1**. The sequences were aligned on the MultAlin web server (Corpet, 1988) (http://multalin.toulouse.inra.fr/multalin/, accessed 9/2/2022). Nucleotide substitutions per site (K_0_) were calculated by the formula of Kimura (1980) based upon pairwise alignments of sequences with all indels removed. Indels per site (I) were calculated as the number of indels in a pairwise alignment divided by the number of nucleotides in the alignment with indels removed (Laroche et al., 1997). Intron sequences found to differ between congener species were also analyzed for potential restriction fragment polymorphisms with the NEB cutter V 2.0 tool (Vincze et al., 2003) (http://nc2.neb.com/NEBcutter2/index.php, accessed 1/26/2023).

### *In silico* prediction of intron amplification products

Prediction of intron amplification products across a wider range of plant taxa was performed through application of the Primer-BLAST tool (Ye et al., 2012) (https://www.ncbi.nlm.nih.gov/tools/primer-blast/) to selected plant mitochondrial genomes in the NCBI organelle genome database (https://www.ncbi.nlm.nih.gov/genome/organelle/) (both accessed 1/20/2023). Genomes queried included early andiosperms *Magnolia biondii* (NC_049134.1) (Dong et al., 2020) and *Magnolia officinalis* (NC_064401) (unpublished), which could potentially differ in sequence from later diverged andiosperms. Additional orders of monocots were selected to complement the single order (Poales) investigated experimentally. These included *Allium cepa* male-sterilizing (KU318712.1) (Kim et al., 2016) and normal (AP018390.1) (Tsujimura and Terachi, 2018) cytoplasms representing monocot order Asparagales; *Cocos nucifera* (KX028885.1) (Aljohi et al., 2016) representing monocot order Arecacales; and *Zostera japonica* (NC_068803.1) (Chen et al., 2022) and *Zostera marina* (KX808392.1)(Petersen et al., 2017) representing monocot order Alismatales. Also included were dicots *Silene conica* (JF40490.1-JF50629.1), *Silene noctiflora* (KP053825.1-KP053880.1), *Silene latifolia* (HM562727.1) and *Silene vulgaris* (JF750427.1-JF750430.1). *Silene* is an important model genus that includes species exhibiting unusual patterns of mitochondrial genome expansion and nucleotide substitution, which potentially affect primer performance and utility. *Silene conica* and *Silene noctiflora* provide tests of primers on expanded mitochondrial genomes that exhibit accelerated nucleotide substitution rates in comparison to *Silene latifolia* and *Silene vulgaris* (Sloan et al., 2012).

## Results

### Mitochondrial intron amplification across angiosperm taxa

**Figure 1:**
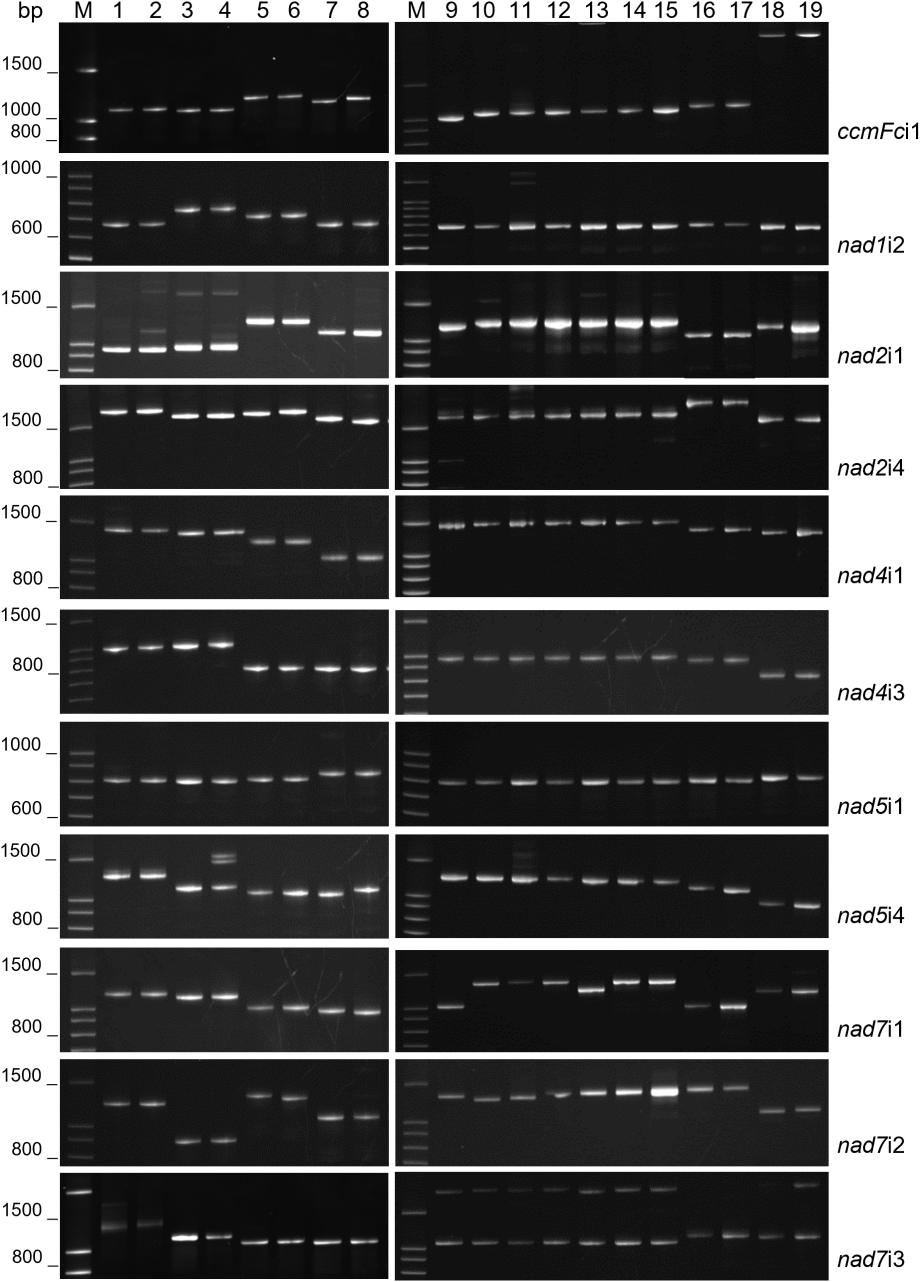
Mitochondrial intron lengths vary between but are conserved within plant genera. PCR amplification products of 11 plant mitochondrial introns were analyzed by polyacrylamide gel electrophoresis. M corresponds to a 100 base pair (bp) DNA ladder. DNA templates for PCR were as follows: 1) *Solanum pinnellii* LA716, 2) *Solanum lycopersicon* Bonny Best, 3) *Raphanus sativus* Red Velvet, 4) *Raphanus sativus* April Cross, 5) *Cynodon dactylon* Royal Cape, 6) *Cynodon transvaalensis* Frankenwald Fine, 7) *Cenchrus americanus* Tifleaf3, 8) *Cenchrus purpureus* Merkeron, 9) *Poncirus trifoliata* English Large Flower, 10) *Citrus japonica* Meiwa, 11) *Citrus medica* Etrog, 12) *Citrus maxima* Hirado Buntan, 13) *Citrus reticulata* Ponkan, 14) *Citrus paradisi* Ruby Red, 15) *Citrus sinensis* Valencia, 16) *Vaccinium virgatum* Tifblue, 17) *Vaccinium corymbosum* Blue Crop, 18) *Phaseolus vulgaris* Calima, 19) *Phaseolus vulgaris* Jamapa.

The intron primer sets (**Table 2**) successfully amplified the target intron in each of the 19 entries investigated (**Figure 1**, **Table 3**). PCR reactions generally produced a single major product, although additional products of low abundance were detected for some *nad2*i1, *nad5*i4 and *nad7*i3 amplifications (**Figure 1**).

**Table 3:** Intron PCR product length^a^ estimated by Advance™ capillary electrophoresis.

Entry	*ccmFc*i1	*nad1*i2	*nad2*i1	*nad2*i4	*nad4*i1	*nad4*i3	*nad5*i1	*nad5*i4	*nad7*i1	*nad7*i2	*nad7*i3
*S. pennellii* LA716^b^	1041	632	1133	1680	1436	751	900	1283	975	1295	1205
*S. lycopersicum* Bonny Best^b^	1048	644	1136	1682	1441	757	898	1290	955	1307	1205
*P. vulgaris* Calima^c^	4284	630	1408	1806	1458	739	926	1087	954	1212	1153
*P. vulgaris* Jamapa^c^	4312	638	1411	1806	1452	743	918	1090	963	1200	1160
*V. corymbosum* Blue Crop^d^	1072	649	1262	1858	1446	737	901	1302	866	1377	1139
*V. virgatum* Tifblue^d^	1067	644	1254	1847	1424	742	890	1299	865	1376	1142
*R. sativus* Red Velvet^e^	1064	673	1083	1896	1475	742	892	1154	1048	898	1134
*R. sativus* April Cross^e^	1061	678	1079	1899	1476	742	895	1167	1055	916	1132
*C. dactylon* PI290868^f^	1122	610	1375	1621	1278	676	926	1088	941	1109	1086
*C. transvaalensis* PI290695^f^	1116	606	1364	1558	1272	678	925	1099	933	1124	1080
*C. americanus* Tifleaf3^g^	1098	608	1358	1512	1009	675	912	1084	916	1134	1073
*C. purpureus* Merkeron^g^	1092	606	1363	1543	995	675	919	1030	937	1126	1076
*P. trifoliata* English Large^h^	1041	641	1262	1847	1468	758	886	1233	943	1421	1145
*C. japonica* Meiwa^h^	1085	643	1301	1842	1475	755	892	1238	977	1414	1158
*C. medica* Etrog^h^	1078	642	1274	1858	1484	755	890	1236	950	1418	1150
*C. maxima* Hirado Buntan^h^	1078	644	1302	1858	1477	755	893	1236	969	1424	1173
*C. reticulata* Ponkan^h^	1071	641	1322	1874	1485	755	892	1240	944	1433	1153
*C. paradisi* Ruby Red^h^	1077	648	1302	1853	1470	755	892	1235	959	1423	1157
*C. sinensis* Valencia^h^	1076	645	1298	1864	1475	753	893	1241	952	1437	1167
Range	1041 -4312	606 - 678	1083 -1411	1512 - 1899	995 - 1485	675 - 758	886 - 926	1030 - 1299	865 - 1055	898 - 1437	1073 - 1205

a PCR product sizes reported in DNA nucleotide pairs are the means of two biological replicates, or two technical replicates for *C. dactylon* and *C. transvaalensis*.

b Genus *Solanum* representing Eudicot order Solanales.

c Genus *Phaseolus* representing Eudicot order Fabales; Calima and Jamapa representing the Andean and Mesoamerican gene pools, respectively.

d Genus *Vaccinium* representing Eudicot order Ericales.

e Genus *Raphanus* representing Eudicot order Brassicales.

f Genus *Cynodon* representing Monocot order Poales.

g Genus *Cenchrus* representing Monocot order Poales.

h Genus *Poncirus* or *Citrus* representing Eudicot order Sapindales.

The increasing number of complete plant mitochondrial genome sequences enabled investigation of the potential for these primer sets to amplify target introns in additional taxa. Primer-BLAST analysis of selected fully sequenced mitochondrial genomes predicted successful application of the introns in early angiosperms represented by *Magnolia biondii* and *Magnolia officionalis*; additional orders of monocots represented by *Allium cepa*, *Cocos nucifera*, *Zoster japonica* and *Zoster marina*; and an additional order of dicots represented by *Silene conica*, *Silene latifolia*, *Silene noctifolora* and *Silene vulgaris* (**Table 4**). Of the 121 primer-accession combinations tested, 79 predicted a single amplification product produced by perfectly matched primers. An additional 18 combinations predicted a single amplification product produced by primers with only one or two mis-matched nucleotides between the target genome and primer set. The 11 primer sets are therefore predicted to be useful for the amplification of mitochondrial introns across the angiosperms. Primers were predicted to be less effective for plant mitochondrial genomes that exhibit exceptionally high rates of genome expansion and nucleotide substitution. *Silene conica* and *Silene noctiflora* represent expanded mitochondrial genomes with accelerated nucleotide substitution rates in comparison to *Silene latifolia* and *Silene vulgaris* (Sloan et al., 2012). While all primer sets were predicted to amplify single products in *Silene latifolia* and *Silene vulgaris*, most primer sets predicted multiple, weak matches to *Silene conica* and *Silene noctiflora*. Nevertheless, 3-4 primer sets were still predicted to work well for these two templates (**Table 4**).

**Table 4:** Intron PCR product length predicted by Primer-BLAST^a^.

Entry	*ccmFc*i1	*nad1*i2	*nad2*i1	*nad2*i4	*nad4*i1	*nad4*i3	*nad5*i1	*nad5*i4	*nad7*i1	*nad7*i2	*nad7*i3
*Allium cepa* ^b^ CMS-S	1142	588	1576	1621	1336	1988	903	1266	1382	958	1301
*Allium cepa* ^b^ Normal	1142	596	1576	1611	1336	1988	903	1266	1410	958	1301
*Cocos nucifera* ^c^	1080	624	1313 2067^d^	1552	1358	2368	916	1072	924	1579	1056
*Magnolia biondii* ^e^	1133 1112	626	1433	1569 1332^d^	1380	2391	891	1400	925	1532 And MWT^f^	1059
*Magnolia officinalis* ^e^	1151	632	1451	1584 and MWT	1380	2437	901	1430	938 1490^d^	1563 433^d^	1079
*Silene conica* ^g^ isolate ABR	1055 1105^d^	MWT	1028	1273 3712^d^	3165^d^	1674	894	1162 2659^d^	1534 and MWT	565 1245^d^	837 2254^d^
*Silene latifolia* alba	1067	655	1131	1405	1500	1951	906	1136	1003	800	1173
*Silene noctiflora* ^g^ isolate BRP	NM^h^	MWT	1058 1123 2491^d^	MWT	1546 and MWT	707	920	1151	889	653 and MWT	NM^h^
*Silene vulgaris* isolate SD2	1067	672	1136	1409	1484	1975	924	1146	989	798	1169
*Zostera japonica* ^i^	1621	552	1454	1808	1264	1456	993	1311	1283	788	2011
*Zostera marina* ^i^	1937	549	1515 2640^d^	2128	1224	1456	999	1260	1283 1505^d^	788	2287

a PCR product sizes in DNA nucleotide pairs were predicted by NCBI Primer BLAST < https://www.ncbi.nlm.nih.gov/tools/primer-blast/> (accessed 1/23/2023). Numbers without superscripts indicate predicted PCR products with primers having 0-2 mismatches per primer on the target template.

b *Allium cepa* male-sterilizing (KU318712.1) and normal (AP018390.1) cytoplasms representing monocot order Asparagales with the male sterilizing cytoplasm of inter-specific origin (Manjunathagowda et al., 2021).

c *Cocos nucifera* (KX028885.1) representing monocot order Arecacales.

d Single weak target with 4 or 5 template mismatches per primer.

e *Magnolia bondii* (NC_049134.1) and *Magnolia officinalis* (NC_064401) representing early angiosperm Magnoliales.

f MWT, multiple weak targets with 4-5 template mismatches per primer.

g *Silene conica* (JF40490.1-JF50629.1) and *Silene noctiflora* (KP053825.1-KP053880.1) exhibit expanded genomes and accelerated nucleotide substitution rates in comparison to Silene latifolia (HM562727.1) and Silene vulgaris (JF750427.1-JF750430.1) (Sloan et al., 2012).

h No match to template.

i *Zostera japonica* (NC_068803.1) and *Zostera marina* (KX808392.1) representing monocot order Alismatales.

### Intron length polymorphisms

**Figure 2:**
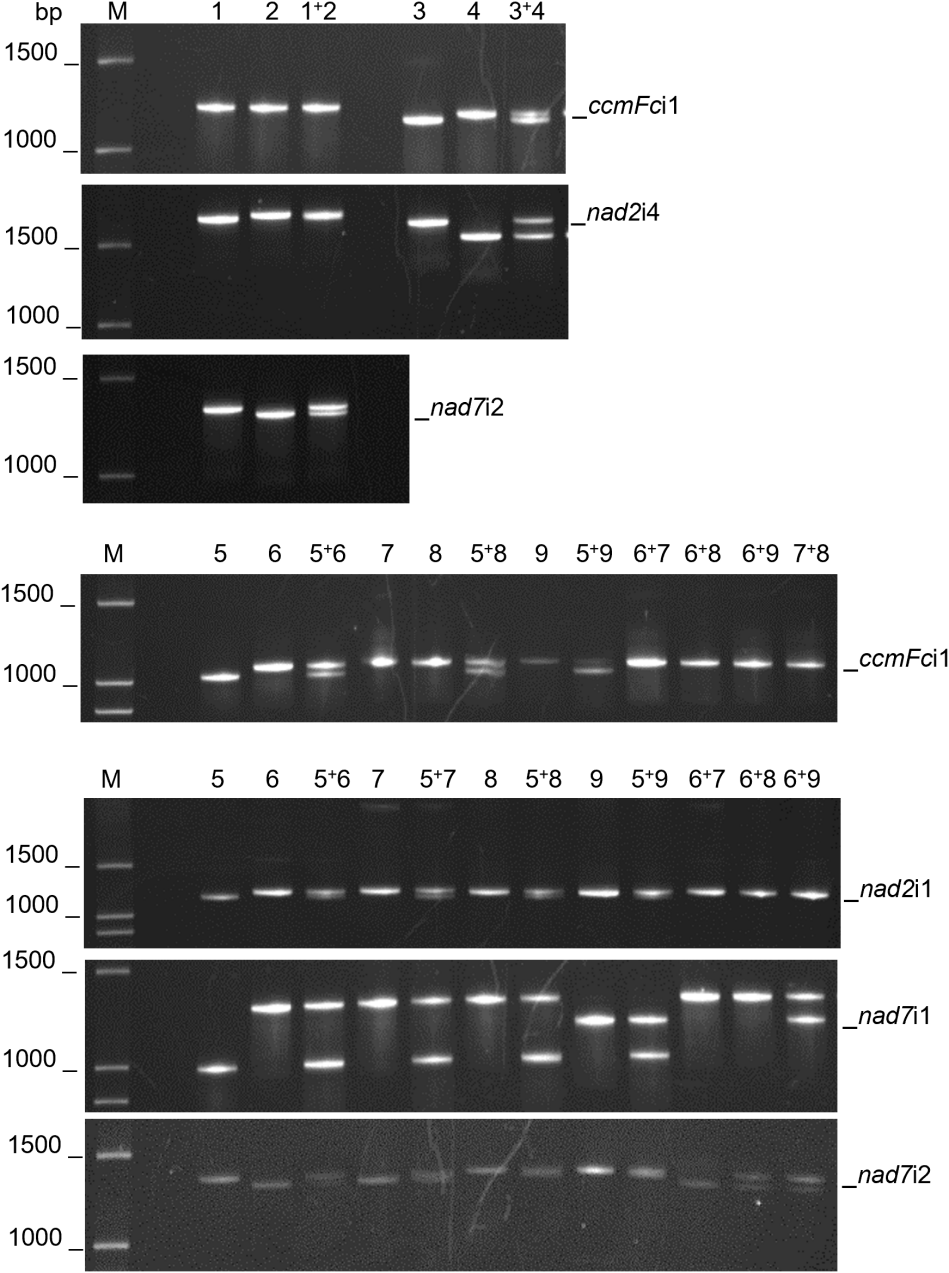
Mitochondrial intron length polymorphisms that distinguish related species. PCR amplification products of mitochondrial introns were separated by polyacrylamide gel electrophoresis. PCR products were analyzed individually and as mixtures to confirm the indel polymorphisms. M corresponds to a 100 base pair (bp) DNA ladder. DNA templates for PCR were as follows: 1) *Cynodon dactylon* Royal Cape, 2) *Cynodon transvaalensis* Frankenwald Fine, 3) *Cenchrus americanus* Tifleaf3, 4) *Cenchrus purpureus* Merkeron, 5) *Poncirus trifoliata* English Large Flower, 6) *Citrus japonica* Meiwa, 7) *Citrus medica* Etrog, 8) *Citrus maxima* Hirado Buntan, 9) *Citrus reticulata* Ponkan. Polymorphisms between *Cenchrus* spp. were confirmed for *ccmFc*i1 and *nad2*i4 and between *Cynodon* spp. for *nad7*i2. *CcmFc*i1, *nad2*i1, and *nad7*i1 polymorphisms differentiated *P. trifoliata* (5) from *Citrus* species (7-9). *Nad7*i1 also distinguished *C. reticulata* (9) from *C. japonica*, *C. medica* and *C. maxima* (6-8), whereas *nad7*i2 polymorphisms distinguished *P. trifoliata*, *C. maxima* and *C. reticulata* (5, 8, 9) from *C. japonica* and *C. medica* (6, 7).

The fractionation of experimentally produced intron amplification products by gel electrophoresis (**Figure 1**) and AdvanCE™ FS96 capillary electrophoresis (**Table 3**) demonstrated significant intron length variation among diverse angiosperm genera, in agreement with primer-BLAST observations (**Table 4**). Intron lengths, as estimated by the AdvanCE™ capillary technique, varied across genera by as few as 40 nucleotides in the case of *nad5*i1 to as many as 539 nucleotides in the case of *nad7*i2. This was excluding the extreme size (4284 nucleotides) of *Phaseolus ccmFc*i1, which likely reflects a split intron. Length polymorphisms between congener species were, however, few in number and challenging to detect by electrophoresis. The well-to-well variation of the AdvanCE™ FS96 precluded use of length values to detect small indel polymorphisms in relatively large DNA amplification products. Length polymorphisms were identified by fractionation of amplification products on polyacrylamide gels (**Figure 1**) and confirmed by acrylamide gel electrophoresis of PCR product mixtures (**Figure 2**) for congeners of *Cenchrus* (*ccmFc*i1 and *nad2*i4), *Cynodon* (*nad7*i2) and *Citrus* (*nad7*i1 and *nad7*i2) species. Intron length polymorphisms are summarized in **Table 5**. The three *Citrus* maternal lineages and *C. japonica* were individually distinguished by the combination of *nad7*i1 and *nad7*i2 polymorphisms. *C. paradisi* (grapefruit) and *C. sinensis* (orange) were not distinguished from their respective *C. maxima* and *C. reticulata* maternal lineages. *Citrus* species were distinguished from *P. trifoliata* by length polymorphisms in *ccmFc*i1, *nad*2i1, *nad7*i1, and *nad7*i2. Electrophoresis did not, however, distinguish the introns of the two *Vaccinium* or *Solanum* species, *Phaseolus* gene pools, or *Raphanus sativus* mitotypes.

**Table 5:** Experimentally identified intron length polymorphisms between congener species.

Intron	Polymorphic taxa			
	Allele 1^a^	Allele 2	Allele 3	Allele 4
*CcmFc*i1	*Cenchrus purpureus* (1008)	*Cenchrus americanus* (1004)		
*CcmFc*i1	*Citrus* ssp^b^ (955)	*Poncirus trifoliata* (921)		
*nad2*i1	*Poncirus trifoliata*	*Citrus* ssp^b^		
*nad2*i4	*Cenchrus americanus*	*Cenchrus purpureus*		
*nad5*i4	*Cynodon dactylon* (929)	*Cynodon transvaalensis* (925)		
*nad7*i1	*Citrus maxima* (901) *Citrus japonica* (901)	*Citrus medica* (893)^c^	*Citrus reticulata* (893)^c^	*Poncirus trifoliata* (875)
*nad7*i2	*Cynodon dactylon*	*Cynodon transvaalensis*		
*nad7*i2	*Citrus maxima* *Citrus reticulata* *Poncirus trifoliata*	*Citrus medica* *Citrus japonica*		

a Allele 1 is designated the longest allele. (Intron lengths in nucleotides are indicated for those introns that were sequenced.).

b *Citrus japonica, Citrus medica, Citrus maxima, Citrus reticulata*.

c These entries carried different indels of the same length.

Primer-BLAST demonstrated that short length polymorphisms often distinguish congener species’ mitochondrial introns (**Table 4**). In *Allium*, introns differing by 8, 10 and 28 nucleotides distinguished the male sterilizing cytoplasm, derived by interspecific introgression (Manjunathagowda et al., 2021), from the normal cytoplasm. *Magnolia biondii* and *Magnolia officinalis* varied in seven introns with length differences ranging from 6 to 46 nucleotides. *Silene vulgaris* differed from *Silene latifolia* in nine introns having length variations ranging from 4-24 nucleotides. *Zoster japonica* and *Zoster marina* were polymorphic with respect to length in eight introns. While five of these differences ranged from 3-61 nucleotides, length polymorphisms of 316, 320 and 276 nucleotides were predicted for *ccmFc*i1, *nad2*i4 and *nad7*i3, respectively. DNA sequence information clearly allows detection of mitochondrial intron length polymorphisms that distinguish related plant species.

### Intron sequence analysis

*CcmFc*i1, *nad5*i4 and *nad7*i1 introns amplified from 16 entries were sequenced to further characterize indels detected by electrophoresis and to search for additional indels, along with SNPs (**Figures S1**, **S2**, and **S3**, respectively). *Citrus sinensis* (sweet orange with the *C. reticulata* maternal lineage) was not included, and heteroplasmy or seed mixtures in the two commercial *Raphanus sativus* accessions precluded obtaining quality sequences for comparison of these two mitotypes within this species. With respect to intra-species variation, *nad5*i4 and *nad7*i1 sequences did not distinguish the two gene pools of *Phaseolus vulgaris*. The *Phaseolus ccmFc*i1 shared 632 5’ nucleotides and 133 3’ nucleotides with other species separated by a 3353 nucleotide insertion (**Figure S1B**). The two *Phaseolus* accessions were polymorphic for one SNP and a 4 base indel within the 3353 nucleotide insertion, but were not polymorphic with respect to the intron regions. Moreover, the three *C. paradisi* introns were not polymorphic with respect to those of their *C. maxima* maternal ancestor. Sequencing further characterized indels detected by gel electrophoresis and revealed additional length polymorphisms (**Table 5**). The *ccmFc*i1 length polymorphism differentiating *P. trifoliata* from *Citrus* entries was due to separate deletions of 8, 9, and 17 nucleotides in *P. trifoliata* compared to *Citrus* (**Figure S1A**). Similarly, the polymorphism in *nad7*i1 was caused by separate deletions of 9, 8, and 9 nucleotides in *P. trifoliata* relative to *C. maxima, C. medica* and *C. japonica. C. reticulata* shared the 8 nucleotide deletion with *P. trifoliata*, while *C. medica* carried a unique 8 nucleotide deletion (**Figure S3**). The *ccmFc*i1 sequence distinguishing *Cenchrus* congeners was a 4 nucleotide indel (**Figure S1A**). Additional length polymorphisms identified by sequencing included a 4 nucleotide *nad5*i4 indel that distinguished *Cynodon* congeners (**Figure S2**) and a 4 nucleotide *nad*7i1 that distinguished *Cenchrus* congeners (**Figure S3**). Sequencing did not reveal indel polymorphisms between *Vaccinium* or *Solanum* congeners.

Sequences of three introns identified only nine SNPs that distinguished congener species (**Table 6**). The *nad5*i4 sequence alignment (**Figure S2**) revealed a SNP that distinguished *V. corymbosum* from *V. virgatum*. This was the only *Vaccinium* polymorphism identified in this study. Three *nad5*i4 SNPs distinguished *C. dactylon* from *C. transvaalensis* (**Figure S2**). In addition to the *nad7*i1 indels, a *nad7*i1 SNP was found to distinguish *C. reticulata* from other *Citrus* species (**Figure S3**). Of the nine SNPs, only one was a C/T difference that could possibly be erased at the RNA level by plant mitochondrial C-to-T RNA editing. In these comparisons, the frequency of SNPs per site (K_0_) within genera was low - zero in the case of *ccmFc*i1. The average K_0_ for *nad5*i4 and *nad7*i1 in congeneric species comparisons was 0.03 and 0.01, respectively, that of comparisons among dicot genera (**Table 7**). The frequency of indels per site (I) within genera was also low, 0.02-0.10 of I for comparisons among dicot genera (**Table 7**).

**Table 6:** Intron nucleotide^a^ polymorphisms between congener species.

Intron	Polymorphic taxa		
	Allele 1	Allele 2	Allele 3
*nad5*i4	*Citrus maxima* 753 A 857 G	*Citrus reticulata* ^b^ 753 A 857 T	*Citrus medica* 753 C 857 G
*nad5*i4	*Vaccinium corymbosum* 982 G	*Vaccinium virgatum* 982 T	
*nad5*i4	*Cynodon dactylon* 659 G 660 T 662 T	*Cynodon transvaalensis* 659 T 660 C 662 A	
*nad7*i1	*Citrus reticulata* 787 C	*Citru*s ssp^c^ 787 A	
*nad7*i1	*Cenchrus americanus* 764 A	*Cenchrus purpureus* 764 G	

a Nucleotides are numbered according to the multitaxa alignments shown in **Figures S1**-**S3**.

b Also *Citrus japonica* and *Poncirus trifoliata*.

c *Citrus maxima, Citrus medica Citrus japonica*, also *Poncirus trifoliata*.

**Table 7:** Average nucleotide substitutions (K_0_) and indels (I) per site within genera and between dicot genera^b^.

Intron	Within genera^a^		Between dicot genera^b^	
	K_0_	I	K_0_	I
*ccmFc*i1	0	0.0001 ± 0.0004	0.036 ± 0.008	0.005 ± 0.003
*nad5*i4	0.0012 ± 0.0009	0.0002 ± 0.0004	0.046 ± 0.006	0.008 ± 0.002
*nad7*i1	0.0002 ± 0.0004	0.0008 ± 0.0009	0.028 ± 0.003	0.007 ± 0.001

a Mean values ± standard deviation calculated for seven pair-wise species comparisons: *Citrus maxima - Citrus reticulata, Citrus maxima - Citrus medica, Citrus medica - Citrus reticulata, Cynodon dactylon - Cynodon transvaalensis, Cenchrus americanus - Cenchrus purpureus, Solanum lycopersicum - Solanum pennellii, Vaccinium corymbosum - Vaccinium virgatum*.

b Mean values ± standard deviation calculated for pair-wise species comparisons: *Citrus maxima - Solanum lycopersicum, Citrus maxima - Vaccinium corymbosum*, and *Solanum lycopersicum - Vaccinium corymbosum*.

While sequence analysis is the most direct means of identifying length and SNP variation in amplified introns, these polymorphisms also create restriction pattern differences. Analysis of sequenced introns with NEB Cutter (**Table S1**) associated unique restriction patterns with the variant alleles reported in **Tables 5** and **6**. The only exception was the SNP that distinguished *Vaccinium corymbosum* and *Vaccinium virgatum nad5*i4 created no RFLPs across the 112 enzymes predicted by the NEB Cutter tool to cut these templates.

## Discussion

### Universal primers for amplification of plant mitochondrial introns

The 11 PCR primer sets used in this work demonstrated robust amplification of the target mitochondrial introns across 16 species representing eight plant genera and seven plant orders. Primer-BLAST analysis with these same primer sets predicted successful amplification of mitochondrial introns from early angiosperms and additional orders of monocots and dicots. This expands and improves the available universal primers for plant mitochondrial introns (Demesure et al., 1995; Dumolin-Lapegue et al., 1997; Duminil et al., 2002). Aleksić (2016) found limited applicability of previously developed universal mitochondrial primers to legume (*Fabaceae*) species and suggested family-specific primers as a more practical approach. The primer sets employed here successfully amplified the mitochondrial introns of *P. vulgaris* as a representative legume. Previous universal primer design strategies (Duminil et al., 2002; Froelicher et al., 2011) utilized mitochondrial sequences conserved between *A. thaliana* and *B. vulgaris* only. Primer design based on conserved introns and flanking sequences from seven plant species (Grosser, 2011) likely contributed to the extended applicability of the current primer sets. Although most plant species’ mitochondrial genomes evolve slowly with respect to coding sequences (Wolfe et al., 1987; Palmer and Herbon, 1988), plant genera containing taxa with widely varying rates of mitochondrial nucleotide substitution have been identified (Cho et al., 2004; Parkinson et al., 2005; Mower et al., 2007; Sloan et al., 2009). Primer-BLAST analysis did predict that some, but not all, of the 11 primer sets would work reliably on the *Silene* species having rapidly evolving mitochondrial coding sequences. A further complication with *Silene conica* and *Silene noctiflora* is that their highly expanded genomes apparently contain multiple, degenerate targets for the intron flanking primers (**Table 4**).

### Intron polymorphism between and within genera

Mitochondrial intron length polymorphisms detectable by electrophoretic techniques were frequently observed between genera, whereas comparisons within genera revealed primarily short intron length variations. Large indels are therefore tolerated within introns, but rates of such variation are low within genera. These contrasting observations likely reflect the evolutionary processes that shaped modern plant organellar group II introns from their self-splicing, progenitor introns. On the one hand, altered intron sequences combined with novel nuclear and organelle-encoded splicing factors to maintain competence for splicing while shifting away from the group II ribozymic, self-splicing structures (Bonen, 2008; Brown et al., 2014). At the same time, the requirement for splicing factors to evolve in concert with the intron structure likely constrained variants that can be successfully spliced (de Longevialle et al., 2010; Zimmerly and Semper, 2015). Plant organelle introns retain significant common structural features (Bonen, 2008). Moreover, they reside within genes essential to photosynthesis or respiration, creating selective pressure for the maintenance of efficient splicing (Brown et al., 2014; Zimmerly and Semper, 2015; Best et al., 2020). Arrays of protein factors are required for the splicing of plastid and mitochondrial introns. These include members of the maturase family, descended from the maturases encoded in ribozymic, self-splicing group II introns (Schmitz-Linneweber et al., 2015), along with APO, CRM, PORR, PPR and TERF families of RNA binding proteins. With the exception of one plastid and one mitochondria-encoded maturase, these proteins are encoded by the nuclear genome and imported into the organelles where they act in combinatorial fashion for the splicing of particular introns or groups of introns (de Longevialle et al., 2010; Brown et al., 2014; Zimmerly and Semper, 2015; Wang et al., 2022). The complexity and specificity of this process may explain the lack of large intron indels found within genera.

The plant mitochondrial intron length differences between congener species as predicted by by Primer-BLAST averaged 17 nucleotides, excepting the three *Zoster* introns with larger differences. The experimentally characterized indels that distinguished congeners or cross-compatible species averaged less than 10 nucleotides in length, necessitating high-resolution acrylamide gels or DNA sequencing for discernment. Gel-resolved intron length polymorphisms differentiated *Citrus*, *Cenchrus*, and *Cynodon* congeners, but intron sequencing provided a more accurate picture of indel polymorphisms. The *nad7*i1 amplicons of *C. medica* and *C. reticulata*, for example, carried different indels of the same length. Even when larger intron size differences were apparent within genera, sequencing revealed them to result from multiple short indels. This is consistent with prior reports that short indels (1-10 bp) comprise greater than 50% of indels in plant mitochondrial introns and probably originate from slipped strand mispairing events during replication (Laroche et al., 1997).

For each of the three introns sequenced in the present study, the average frequency of nucleotide substitutions per site (K_0_) was also low within genera, but SNPs that distinguished congeners of *Citrus*, *Cynodon*, *Cenchrus*, and *Vaccinium* were identified. These can serve as useful markers through workflows such as cleaved amplified polymorphic sequence (CAPS), PCR combined with sequencing, or amplification refractory mutation analysis strategies (Lo, 1998; Ciarmiello et al., 2013). With one exception, SNPs and short indels that distinguished congeners’ introns also created CAPS markers (**Table S1**). More broadly, K_0_ values for comparisons between *Citrus*, *Vaccinium* and *Solanum* as representative dicot genera (**Table 7**) were similar to those reported by Laroche et al. (1997) for comparisons of six mitochondrial introns between two to three dicot genera. In the present study, K_0_ values for intron sequence comparisons between congeneric species were 0.01-0.03 times those for comparisons between dicot genera. The low nucleotide substitution rates likely result from the low frequency of nucleotide substitutions characteristic of most plant mitochondrial genomes, typically three to ten times lower than nuclear nucleotide substitution rates (Wolfe et al., 1987; Palmer and Herbon, 1988; Drouin et al., 2008).

The limited variation of mitochondrial introns within plant genera contrasts with the extensive diversity of nuclear introns, which show a high frequency of length and substitution polymorphisms within species of *Oryza* (Wang et al., 2005), *Solanum* (Wang et al., 2010), *Allium* (Jayaswal et al., 2019) and *Medicago* (Shilpa and Lohithaswa, 2021) among others. While no mitochondrial intron polymorphisms distinguished *Solanum lycopersicum* from *S. pennellii*, two studies document extensive nuclear intron polymorphisms within *Solanum lycopersicum* (Van Deynze et al., 2007; Wang et al., 2010). Organellar group II introns are considered the ancestors of nuclear introns, which lack folding constraints because they share the use of spliceosomal RNAs that have taken on the functions of the group II intron domains (Sharp, 1991). The spliceosome is a complex that is highly malleable in order to accommodate diverse exon ends and alternative splicing, perhaps permitting more varied intron sequences (Chen and Moore, 2014).

### Application of plant mitochondrial intron polymorphisms

When present, organelle intron polymorphisms have valuable applications for determining inheritance in sexual crosses or somatic hybridizations. The markers investigated in this study have proved useful for determining organelle inheritance in *Citrus* cybrids. Cybrids are produced spontaneously as a by-product of protoplast fusion and are characterized by the diploid nuclear genome of the mesophyll fusion partner, the mitochondrial genome of the embryogenic callus partner, and random inheritance of chloroplast DNA (Grosser et al., 1996; Cabasson et al., 2001; Guo et al., 2004; Guo et al., 2013). This contrasts with typical protoplast fusion products, which possess tetraploid nuclei inherited from both parents. Cybrids provide a means to quickly create novel combinations of nuclear and organellar genotypes and to evaluate their phenotypic consequences. Specific organelle genotypes are associated with beneficial traits in cybrids. For example, grapefruit cybrids with mandarin mitochondrial DNA exhibit an extended season of high-quality fruit (Satpute et al., 2015), whereas grapefruit cybrids with kumquat plastid DNA exhibit increased resistance to citrus canker regardless of mitochondrial origin (Omar et al., 2017). The *nad7*i1 and *nad7*i2 primer sets (**Table 2**) were utilized, respectively, for verification and characterization of mitochondrial DNA inheritance in these two sets of cybrids, illustrating application for mitochondrial intron markers.

The currently reported *nad7*i1 marker overlaps with and confirms one of the three markers that Froelicher et al. (2011) demonstrated to be polymorphic in *Citrus*. Because our primer set amplified the entire intron, a new SNP was added to the previously published indels. Moreover, the list of intron markers polymorphic for *Citrus* species was expanded to include *nad7*i2, *ccmFc*, and *nad2*i1. The additional polymorphic markers did not, however, further distinguish differences within the seven citrus mitotypes identified by Froelicher et al. (2011). For example, *C. maxima* and its maternal derivative *C. paradisi* remained indistinguishable for all introns sequenced in this study.

Due to the lack of conserved gene order among plant mitochondrial genomes, often even between closely related taxa, assembling plant mitochondrial genome sequences presents special challenges and can preclude the universal application of intergenic sequences for distinguishing between closely related groups (Duminil and Besnard, 2021). Mitochondrial intron markers have demonstrated applicability in studies of population genetics, genotype characterization, detection of past hybridizations, and biogeographic studies of gene pool distributions (Ciarmiello et al., 2013; Aizawa et al., 2014; Xiang et al., 2014; Kersten et al., 2015). The current study documents a widely applicable set of primers for the mitochondrial marker toolbox and provides insights into the conservation and variation of plant mitochondrial introns.

## Data availability statement

The datasets presented in this study can be found in online repositories. The names of the repository/repositories and accession number(s) can be found below: https://www.ncbi.nlm.nih.gov/genbank/, OP800658-OP800705.

## Author contributions

CC, FG, JGro, and JGra: designed the study and collected the genetic materials. KC, KL, MG, SS, MM and YL: conducted the research. CC and MG: wrote the manuscript. All authors contributed to the article and approved the submitted version.
