# Supplementary material for: Plant mitochondrial introns as genetic markers - conservation and variation
Source: Front Plant Sci. 2023 Mar 20;14:1116851. doi: 10.3389/fpls.2023.1116851 (PMC10067590; doi:10.3389/fpls.2023.1116851)
Supplement: Supplementary file 1 [file Table_1.pdf]

Table S1. NEB Cutter predicted restriction fragment length polymorphisms among sequenced introns[a]

Intron-enzyme	Polymorphic Restriction Fragment Pattern[b]			
*ccmF*ci1- BccI	*Cenchrus americanus*<br>3, 108, 139, 182, 572	*Cenchrus purpureus*<br>3, 108, 139, 186, 572		
*ccmF*ci1 - BstXI	*Citrus* ssp.<br>421, 534	*Poncirus trifoliata*<br>921		
*Nad5*i4 - AluI	*Citrus maxima*<br>*Citrus medica*<br>96, 122, 135, 704	*Citrus reticulata*<br>*Citrus japonica Poncirus trifoliata*<br>96, 122, 135, 426, 278		
*Nad5*i4 - BbsI	*Cynodon dactylon*<br>452, 477	*Cynodon transvaalensis*<br>925		
*Nad5*i4 - BsRI	*Cynodon dactylon*<br>473, 456	*Cynodon transvaalensis*<br>925		
*Nad5*i4 - BstI	*Cynodon dactylon*<br>23, 81, 848	*Cynodon transvaalensis*<br>23, 77, 848		
*Nad5*i4 - BtsCI	*Cynodon dactylon*<br>48, 192, 689	*Cynodon transvaalensis*<br>925		
*Nad5*i4 - FokI	*Cynodon dactylon*<br>41, 179, 709	*Cynodon transvaalensis*<br>925		
*Nad7*i1 - BceAI	*Cenchrus americanus*<br>873	*Cenchrus purpureus*<br>184, 693		
*Nad7*i1 - BsrDI	*Cenchrus americanus*<br>168, 705	*Cenchrus purpureus*<br>877		
*Nad7*i1 - Bpu10I	*Citrus maxima*<br>107, 973	*Citrus medica*<br>893	*Citrus reticulata*<br>107, 786	*Poncirus trifoliata*<br>107, 768
*Nad7*i1 - DdeI	*Citrus maxima*<br>107, 973	*Citrus medica*<br>893	*Citrus reticulata*<br>107, 786	*Poncirus trifoliata*<br>107, 768
*Nad7*i1 - BanII	*Citrus maxima*<br>545, 356	*Citrus medica*<br>537, 356	*Citrus reticulata*<br>545, 348	*Poncirus trifoliata*<br>875

[a]Sequenced introns containing SNP or indel polymorphisms were analyzed on the NEB Cutter version 2.0 website (Vincze et al., 2003) < http://nc2.neb.com/NEBcutter2/index.php > accessed 1/26/2023.

[b]Predicted restriction fragment patterns that reveal polymorphisms distinguishing related taxa are reported nucleotide pairs.
