# Supplementary material for: Plant mitochondrial introns as genetic markers - conservation and variation
Source: Front Plant Sci. 2023 Mar 20;14:1116851. doi: 10.3389/fpls.2023.1116851 (PMC10067590; doi:10.3389/fpls.2023.1116851)
Supplement: Supplementary file 2 [file Image_1.pdf]

A)

```
              1                                                 50
       Cm     TGCGACCTGG CGGCTTATGT GCGACCTGTG GCCCACGCCT CCTATT....
       Pt     TGCGACCTGG CGGCTTATGT GCGACCTGTG GCCCACGCCT CCTATT....
       Sl     TGCGACCCGG CGGCTTATGT GCGACCTGTG GCCCACGCCT CCTATT....
       Vc     TGCGACCCGG CGGCTTATGT GCGACCTGTG GCCCACGCCT CCTATT....
       Cd     TGCGACCCGA TGGCTTATGT GCGACCTGTG GCCCACGCCT CCTATTCTAT
       Ca     TGCGACCCGA TGGCTTATGT GCGACCTGTG GCCCACGCCT CCTATTCTAT
       Cp     TGCGACCCGA TGGCTTATGT GCGACCTGTG GCCCACGCCT CCTATTCTAT
Consensus     TGCGACCcGg cGGCTTATGT GCGACCTGTG GCCCACGCCT CCTATT....

              51                                               100
       Cm     .TGTT..... CAGGGCGGGC GGCGTGAACT CTGATTCGAT CCGGGTATTC
       Pt     .TGTT..... CAGGGCGGGC GGCGTGAACT CTGATTCGAT CCGGGTATTC
       Sl     .TGTT..... CAGGGCGGGC GGCGTGAACT CTGATTCGAT CCGGGTATTC
       Vc     .TGTT..... CAGGGCGGGC GGCGTGAACT CTGATTCGAT CCGGGTATTC
       Cd     TTGTT..... CAGGGCGGGC GGCGTGAACT CTGATTCGAT CCGGGTATTC
       Ca     TTGTTTTGTT CAGGGCGGGC GGCGTGAACT CTGATTCGAT CCGGGTATTC
       Cp     TTGTTTTGTT CAGGGCGGGC GGCGTGAACT CTGATTCGAT CCGGGTATTC
Consensus     .TGTT..... CAGGGCGGGC GGCGTGAACT CTGATTCGAT CCGGGTATTC

              101                                              150
       Cm     AATCCCGCCA CTGAGATGCT CAGTTGACTC CTTAACCTTG ATAGGAAGAT
       Pt     AATCCCGCCA CTGAGATGCT CAGTTGACTC CTTAACCTTG ATAGGAAGAT
       Sl     AATCCCGCCG CTGAGATGCT CAGTTGACTC CTTAACCTTG ATAGGAAGAT
       Vc     AATCCCGTCG CTGAGATGCT CAGTTGACTC CTTAACCTTG ATAGGAAGAT
       Cd     AATCCCGCCG CTGAGATGCT CAGTTGACTC CTTAACCTTG ATAGGAAGAT
       Ca     AATCCCGCCG CTGAGATGCT CAGTTGACTC CTTAACCTTG ATAGGAAGAT
       Cp     AATCCCGCCG CTGAGATGCT CAGTTGACTC CTTAACCTTG ATAGGAAGAT
Consensus     AATCCCGcCg CTGAGATGCT CAGTTGACTC CTTAACCTTG ATAGGAAGAT

              151                                              200
       Cm     GGCTTATTCA ATAATTCGTG CATAAGGGTA AGGAACTTTG GATGAACTAA
       Pt     GGCTTATTCA ATAATTCGTG CATAAGGGTA AGGAACTTTG GATGAACTAA
       Sl     GGCTTATTCA ATAATTCGTG CATAAGGGTA AGGAACTTTG GATGAACTAA
       Vc     GGCTTATTCA ATAATTCGTG CATAAGGGTA AGGAACTTTG GATGAACTAA
       Cd     GGCTTATTCC AAAATTCGTG CATAAGGGTA AGGAACTTTG GATGAATTAA
       Ca     GGCTTATTCC TAAATTCGTG CATAAGGGTA AGGAACTTTG GATGAATTAA
       Cp     GGCTTATTCC TAAATTCGTG CATAAGGGTA AGGAACTTTG GATGAATTAA
Consensus     GGCTTATTCa atAATTCGTG CATAAGGGTA AGGAACTTTG GATGAAcTAA

              201                                              250
       Cm     TGCGAATGGG TGTAAGCCTC GCTGCTCGGA AACACCCAGT GCTGACCACA
       Pt     TGCGAATGGG TGTAAGCCTC GCTGCTCGGA AACACCCAGT GCTGACCACA
       Sl     TGCGAATGGG TGTAAGCCTC GCTGCTCGGA AACACCCAGT GCTGACCACA
       Vc     TGCGAATGGG TGTAAGCCTC GCTGCTCGGA AACACCCAGT GCTGACCACA
       Cd     TGCGAATGGG TGTAAGCCTC GCAGCTCGGA AACACCCAGT GCTGACCACA
       Ca     TGCGAATGGG TGTAAGCCTC GCAGCTCGGA AACACCCAGT GCTGACCACA
       Cp     TGCGAATGGG TGTAAGCCTC GCAGCTCGGA AACACCCAGT GCTGACCACA
Consensus     TGCGAATGGG TGTAAGCCTC GCtGCTCGGA AACACCCAGT GCTGACCACA

              251                                              300
       Cm     CTGAGAGACA CGAAAGCGCA GGTAACGCCA GTTGGCGAAG TGGTGTTAAG
       Pt     CTGAGAGACA CGAAAGCGCA GGTAACGCCA GTTGGCGAAG TGGTGTTAAG
       Sl     CTGAGAGACA CGAAAGCGCA GGTAACGCCA GTTGGCGAAG TGGCGTTAAG
       Vc     CTGAGAGAGA CGAAAGCGCG GGTAACGCCA GTTGGCGAAG TGGCATTAAG
       Cd     CTGAGAGACA CGAAAGCGCA GGTAATGCCA GTTGGCGAAG TGGCGTTAAG
       Ca     CTGAGAGACA CGAAAGCGCA GGTAATGCCA GTTGGCGAAG TGGCGTTAAG
       Cp     CTGAGAGACA CGAAAGCGCA GGTAATGCCA GTTGGCGAAG TGGCGTTAAG
Consensus     CTGAGAGAcA CGAAAGCGCa GGTAAcGCCA GTTGGCGAAG TGGcgTTAAG
```

```
             301                                                   350
       Cm    CATCCCTAGC GGTACGAAAA GAGAGGTCGT GATGATATCA TCTACGTCCG
       Pt    CATCCCTAGC GGTACGAAAA GAGAGGTCGT GATGATATCA TCTACGTCCG
       Sl    CATCCCTAGC GGTACGAAAA GAGGGGTCGT GATGATATCA TCTACGTCCG
       Vc    CATCCCTAGC GGTACGAAAA GAGAGGTCGT GATGATATCA TCTACGTCCG
       Cd    CATCCCTAGC GGTACGCAAA GAGAGGTCGT GATGATATCA TCTACGTCCG
       Ca    CATCCCTAGC GGTACGCAAA GAGAGGTCGT GATGATATCA TCTACGTCCG
       Cp    CATCCCTAGC GGTACGCAAA GAGAGGTCGT GATGATATCA TCTACGTCCG
Consensus    CATCCCTAGC GGTACGaAAA GAGaGGTCGT GATGATATCA TCTACGTCCG

             351                                                   400
       Cm    TACCGCTCCT CGTGGAGTAG ATCCCGCATC CAACCAAGTC TTT...GACC
       Pt    TACCGCTCCT CGTGGAGTAG ATCCCGCATC CAACCAAGTC TTT...GACC
       Sl    TACCGCTCCT CGTGGAGTAG ATCCCGCATC CAACCAAGTC TTT...GACC
       Vc    TACCGCTCCT CGTGGAGTAG ATCCCGCATC CAACCAAGTC TTT...GACC
       Cd    TACCGCTCCT CGTGGAGTAG ATCCCGCATC CAACCAACCC TTTTTTGACC
       Ca    TACCGCTCCT CGTGGAGTAG ATCCCGCATC CAACCAACCC TTTTTTGACC
       Cp    TACCGCTCCT CGTGGAGTAG ATCCCGCATC CAACCAACCC TTTTTTGACC
Consensus    TACCGCTCCT CGTGGAGTAG ATCCCGCATC CAACCAAgtC TTT...GACC

             401                                                   450
       Cm    AGGGAACGGG AGAATTCCCA CTACCGCTGG CAGGCCAGCC GGGCCGTAAG
       Pt    AGGGAACGGG AGAATTCCCA CTACCGCTGG CAGGCCAGCC GGGCCGTAAG
       Sl    AGGGAACGGG AGAATTCCCA CTACCGCTGG AAGGCCAGCC GGGCCGTGAG
       Vc    AGGGAACGGG AGAATTCCCA CTACCGCTGG CAGGCCAGCC GGGCCGTGAG
       Cd    AGGGAACGGG AGAATTCCCA CTACCGCAGG CAGGCCAGCC GGGCCGTGAG
       Ca    AGGGAACGGG AGAATTCCCA CTACCGCAGG CAGGCCAGCC GGGCCGTGAG
       Cp    AGGGAACGGG AGAATTCCCA CTACCGCAGG CAGGCCAGCC GGGCCGTGAG
Consensus    AGGGAACGGG AGAATTCCCA CTACCGCtGG cAGGCCAGCC GGGCCGTgAG

             451                                                   500
       Cm    CGCGGTGGGA ACGGGCTTCC CAAAAAGCCA GCCCCGGGCC GGGGTCAGCA
       Pt    CGCGGTGGGA ACGGGCTTCC CAAAAAGCCA GCCCCGGGC. .......GCA
       Sl    CGCGGTGGGA ACGGGCTTCC CAAGAAGCCA GCCC.GGGCC GGGGTCAGCA
       Vc    CGCGGTGGGA ACGGGCTTCC CAAAAAGCCA GCCC.GGGCC GGGGTCAGCA
       Cd    CGCGGTGGGA ACGGGCTTCC CAAAAAGCGC GCCCCGGCCC GGG.TCAGCA
       Ca    CGCGGTGGGA ACGGGCTTCC CAAAAAGCGA GCCCCGGCCC GGG.TCAGCA
       Cp    CGCGGTGGGA ACGGGCTTCC CAAAAAGCGA GCCCCGGCCC GGG.TCAGCA
Consensus    CGCGGTGGGA ACGGGCTTCC CAAaAAGCca GCCCcGGgCc ggggtcaGCA

             501                                                   550
       Cm    TAGAATGAAG GGG...ACGG CCCTAATGTT GTGTTGGCAA AGCCAACTTC
       Pt    TAGAATGAAG GGG...ACGG CCCTAATGTT GTGTTGGCAA AGC.......
       Sl    TAGAATGAAG GGG...ACGG CCCTAATCTT GTGTTGGCTT TGCCAACTTA
       Vc    TAGAATGAAG GGG...ACGG CCCTAATGTT GTGTTGGCAA AGCCAACTTC
       Cd    TAGAATGGGG GGGGGGACGG CCCTAATGTT GTGTTGGCC. ..........
       Ca    TAGAATGGGG GGGGGGACGG CCCTAATGTT GTGTTGGCC. ..........
       Cp    TAGAATGGGG GGGGGGACGG CCCTAATGTT GTGTTGGCC. ..........
Consensus    TAGAATGaaG GGG...ACGG CCCTAATgTT GTGTTGGCaa agccaactt.

             551                                                   600
       Cm    TTGGGTTGCG GACGGAGAAG AGCGGACGTG GGGACTCGGG TCGGGGCGCA
       Pt    ..GGGTTGCG GACGGAGAAG AGCGGACGTG GGGACTCGGG TCGGGGCGCA
       Sl    TTGGGTTGCG GGCGGAGAAA AGCGGACGTG GGGACTCGGG TCGGGGCGCA
       Vc    TTGGGTTGCG GGCGGAGAAA AGCGGACGTG GGGACTCGGG TCGGGGCGCA
       Cd    ..GGGTTGCG GGCGGATAAA AGCGGACGTG GGGACTCGGG TCGGGGCACA
       Ca    ..GGGTTGCG GGCGGATAAA AGCGGACGTG GGGACTCGGG TCGGGGCACA
       Cp    ..GGGTTGCG GGCGGATAAA AGCGGACGTG GGGACTCGGG TCGGGGCACA
Consensus    ttGGGTTGCG GgCGGAgAAa AGCGGACGTG GGGACTCGGG TCGGGGCgCA
```

```
           601                                                650
       Cm  GCGTAACTAA GATAGTCATT CCATTTAGGG CGAGACAGAA TGGGCGGGCA
       Pt  GCGTAACTAA GATAGTCATT CCATTTAGGG CGAGACAGAA TGGGCGGGCA
       Sl  GCGTAACTAA GAGAGCCATT CCATTTAGGG CGAGACAAAA TGGGCGGGCA
       Vc  GCGTAACTAA GAGAGCCATT CCATTTAGGG CGAGACAAAA TGGGCGGGCA
       Cd  GCGTAACTAA GAGAGCCATT CTATTTAGTG CGAGACAGAA TGGGCGGGCA
       Ca  GCGTAACTAA GAGAGCCATT CCATTTAGTG CGAGACAGAA TGGGCGGGCA
       Cp  GCGTAACTAA GAGAGCCATT CCATTTAGTG CGAGACAGAA TGGGCGGGCA
Consensus  GCGTAACTAA GAgAGcCATT CcATTTAGgG CGAGACAgAA TGGGCGGGCA

           651                                                700
       Cm  CGAGCGGTCT GGTGTCCAAG CCAATTGGTC AGACGACGAC TACTTCAC..
       Pt  CGAGCGGTCT GGTGTCCAAG CCAATTGGTC AGACGACGAC TACTTCAC..
       Sl  CGGGCGGTCT GGTGTCCGAG CCGATTGGTC AGACGACGAC TACTTCACTT
       Vc  CGAGCGGTCT GGTGTCCGAG CCTATTGGTC AGACGACGAC TACTTCACTT
       Cd  CAAGCGGTCT GGTGTCCGAG CCGATTGGTC AGACGACGAC TACTGCACA.
       Ca  CAAGCGGTCT GGTGTCCGAG CCGATTGGTC AGACGACGAC TACTGCACA.
       Cp  CAAGCGGTCT GGTGTCCGAG CCGATTGGTC AGACGACGAC TACTGCACA.
Consensus  CgaGCGGTCT GGTGTCCgAG CC.ATTGGTC AGACGACGAC TACTtCAC..

           701                                                750
       Cm  .....ATTAG TATTAGCCGC CTATCCCGGA ATGAAATGAG ATGAAATAGA
       Pt  .....ATTAG .......... .......GGA ATGAAATGAG ATGAAATAGA
       Sl  ATTATTTAAG TATTAGCCGC CTATCCCGGA ATGG.....G ATGGAATAGA
       Vc  ATTAGATTAG TATTAGCCGC TTACCCCGGA ATGG.....G ATGGAATAGA
       Cd  .....TATTA TATTAGCCGC CTATCCCGGA ATGGACTGGG ATGGAATAGA
       Ca  .....TATTA TATTAGCCGC CTATCCCGGA ATGGACTGGG ATGGAATAGA
       Cp  .....TATTA TATTAGCCGC CTATCCCGGA ATGGACTGGG ATGGAATAGA
Consensus  .....attag tattagccgc ctatcccGGA ATGga.tg.G ATGgAATAGA

           751                                                800
       Cm  AAGAACGCGA AGC....... .......... .GCT...... ....AGCGC.
       Pt  AAGAACGCGA AGC....... .......... .GCT...... ....AGCGC.
       Sl  AAGAACGCGA AGC....... .......... .GCT...... ....AGCGC.
       Vc  AAGAACGCGA AGC....... .......... .GCT...... ....AGCAC.
       Cd  AAGAACGCGA AGCAACAAGC AAGGGATGAG CGCTTTGTTG CTAAAGCGCA
       Ca  AAGAACGCGA AGCAACAAGC AAGGGATGAG CGCTTTGTTG CTAAAGCGCA
       Cp  AAGAACGCGA AGCAACAAGC AAGGGATGAG CGCTTTGTTG CTAAAGCGCA
Consensus  AAGAACGCGA AGC....... .......... .GCT...... ....AGCgC.

           801                                                850
       Cm  .......... TATAGGATCG GTTT....TT TTGGGGGGAA TAAACTCGCT
       Pt  .......... TATAGGATCG GTTT....TT TTGGGGGGAA TAAACTCGCT
       Sl  .......... TACAGGGTCG GTTT....TT TGGGGGGGAT AG.....GCT
       Vc  .......... TATAGGGTCG GTTT....TT ..GGGGGGAT AA.....GCT
       Cd  TACGTTTTCT TGCTGGGTCG GTTTGTTTTT TGGGGGTGGG GGGCAATAAG
       Ca  TAC....TCT TGCTGGGTCG GTTTGTTTTT TGAGGGTGGG G.......CG
       Cp  TACGTTTTCT TGCTGGGTCG GTTTGTTTTT TGAGGGTGGG G.......CG
Consensus  .......... TataGGgTCG GTTT....TT t.gGGGgGa. .a.....gct

           851                                                900
       Cm  TCTTCTTCTT CACAAGCTTA TCCCCGGCCC C......... ......GACC
       Pt  TCTTCTTCTT CACAAGCTTA TCCCCGGCCC C......... ......GACC
       Sl  TGTGAAGAA. .GCAAGCTTA TCCCCG.CCC C......... ......GACC
       Vc  TGTGAAGAA. .GCAAGTTTA TCCCCG.CCC C......... ......GACC
       Cd  CTTGCTTCTT CACAAGCTTA TCCCCGCCCC CTTTCCTGTC CGTCCCGACC
       Ca  CTTGCTTCTT CGCAAGCTTA TCCCCGCCCC CTTTCCTGTC CGTCCCGACC
       Cp  CTTGCTTCTT CGCAAGCTTA TCCCCGCCCC CTTTCCTGTC CGTCCCGACC
Consensus  t.Tgcttctt cgCAAGcTTA TCCCCG.CCC C......... ......GACC
```

```
           901                                               950
       Cm  GGGAGCAGCA GGGTCTCCCC ATCTTTCCTA AACTTCCCCC GGTCTTCGGC
       Pt  GGGAGCAGCA GGGTCTCCCC ATCTTTCCTA AACTTCCCCC GGTCTTCGGC
       Sl  GGCAGCTGCT AGGTTTCCCC ATCTCTTCTA AACTTCCCCC GGTCTTCGGC
       Vc  GGTAGCTGCT GGGTCTCCCC ATCTCTCCTA AACTTCCCCC GGTCTTCGGC
       Cd  GGCAGCAGTT GGGTCTCCCC ATCTCTCCTC AACTTCCCC. GGTCTTCGGC
       Ca  GGCAGCAGTT GGGTCTCCCC ATCTCTCCTC AACTTCCCC. GGTCTTCGGC
       Cp  GGCAGCAGTT GGGTCTCCCC ATCTCTCCTC AACTTCCCC. GGTCTTCGGC
Consensus  GG.AGCaGct gGGTcTCCCC ATCTcTcCTa AACTTCCCCc GGTCTTCGGC

           951                                              1000
       Cm  CGGAGCTGTA TGAGGCAGAA ACTCGTCCCA CGTACGGTTC GGAGGCCGAG
       Pt  CGGAGCTGTA TGAGGCAGAA ACTCGTCCCA CGTACGGTTC GGAGGCCGAG
       Sl  CCGAGCTGTA TGAGGCAAAA ACTCGTCCCA CGTACGGTTC GGAGGCCGAG
       Vc  CCGAGCTGTA TGAGGCAGAA ACTCGTCTCA CGTACGGTTC GGAGGCCGAG
       Cd  CCGAGCTGTA TGAGGCAGAA AATCGTCCCA CGTACGGTTC GGAGGCCGAG
       Ca  CCGAGCTGTA TGAGGCAGAA ACTCGTCCCA CGTACGGTTC GGAGGCCGAG
       Cp  CCGAGCTGTA TGAGGCAGAA ACTCGTCCCA CGTACGGTTC GGAGGCCGAG
Consensus  CcGAGCTGTA TGAGGCAgAA AcTCGTCcCA CGTACGGTTC GGAGGCCGAG

           1001                               1036
       Cm  CCCCACCCCA GCAGTAATGG TGCGGCTTAG GTCAAC
       Pt  CCCCACCCCA GCAGTAATGG TGCGGCTTAG GTCAAC
       Sl  CCCCACCCCA GCAGTAAGGG TGCGGCTTAG GTCAAC
       Vc  CCCCACCCCA GCAGTAAGAG TGCGGCTTAG GTCAAC
       Cd  CCCCACCCCA GCAATAATGG TGCGGCTTAG GTCAAC
       Ca  CCCCACCCCT GCAATAATGG TGCGGCTTAG GTCAAC
       Cp  CCCCACCCCT GCAATAATGG TGCGGCTTAG GTCAAC
Consensus  CCCCACCCCa GCAgTAAtgG TGCGGCTTAG GTCAAC
```

B)

```
           1                                                 50
 Pv-C      TGCGACCCGG CGGCTTATGT GCGACCTGTG GCCCACGCCT CCTATTTGAT
 Pv-J      TGCGACCCGG CGGCTTATGT GCGACCTGTG GCCCACGCCT CCTATTTGAT
Consensus  TGCGACCCGG CGGCTTATGT GCGACCTGTG GCCCACGCCT CCTATTTGAT

           51                                               100
 Pv-C      TTGTTCAGGG CGGGCGGCGT GAACTCTGAC TCGATCCGGG TATTCAATCC
 Pv-J      TTGTTCAGGG CGGGCGGCGT GAACTCTGAC TCGATCCGGG TATTCAATCC
Consensus  TTGTTCAGGG CGGGCGGCGT GAACTCTGAC TCGATCCGGG TATTCAATCC

           101                                              150
 Pv-C      CGCCGCTGAG ATGCTCAGTT GACTCCTGAA CCTTGATAGG AAGATGGCTT
 Pv-J      CGCCGCTGAG ATGCTCAGTT GACTCCTGAA CCTTGATAGG AAGATGGCTT
Consensus  CGCCGCTGAG ATGCTCAGTT GACTCCTGAA CCTTGATAGG AAGATGGCTT

           151                                              200
 Pv-C      ATTCAATAAT TCGTGCATAA GGGTAAGGAA CTTTGGATGA ACTAATGCGA
 Pv-J      ATTCAATAAT TCGTGCATAA GGGTAAGGAA CTTTGGATGA ACTAATGCGA
Consensus  ATTCAATAAT TCGTGCATAA GGGTAAGGAA CTTTGGATGA ACTAATGCGA

           201                                              250
 Pv-C      ATGGGTGTAA GCCTCGCTGC TCGGAAACAC CCAGTGCTGA CCACACTGAG
 Pv-J      ATGGGTGTAA GCCTCGCTGC TCGGAAACAC CCAGTGCTGA CCACACTGAG
Consensus  ATGGGTGTAA GCCTCGCTGC TCGGAAACAC CCAGTGCTGA CCACACTGAG

           251                                              300
 Pv-C  AGACACGAAA GCGCAGGTAA CGCCAGTTGG CGAAGTGGCG TTAAGCATCC
 Pv-J  AGACACGAAA GCGCAGGTAA CGCCAGTTGG CGAAGTGGCG TTAAGCATCC
Consensus  AGACACGAAA GCGCAGGTAA CGCCAGTTGG CGAAGTGGCG TTAAGCATCC

           301                                              350
 Pv-C      CTAGCGGTAC GAAAAGAGAG GTCGTGATGA TATCATCTAC GTCCGTACCG
 Pv-J      CTAGCGGTAC GAAAAGAGAG GTCGTGATGA TATCATCTAC GTCCGTACCG
Consensus  CTAGCGGTAC GAAAAGAGAG GTCGTGATGA TATCATCTAC GTCCGTACCG

           351                                              400
 Pv-C      CTCCTCGTGG AGTAGATCCC GCATCCAACC AAGTCTTTGA CCAGGGAACG
 Pv-J      CTCCTCGTGG AGTAGATCCC GCATCCAACC AAGTCTTTGA CCAGGGAACG
Consensus  CTCCTCGTGG AGTAGATCCC GCATCCAACC AAGTCTTTGA CCAGGGAACG

           401                                              450
 Pv-C      GGAGAATTCC CACTACCGCT GGAAGGCCAG CCGGGCCGTG AGCGCGGTGG
 Pv-J      GGAGAATTCC CACTACCGCT GGAAGGCCAG CCGGGCCGTG AGCGCGGTGG
Consensus  GGAGAATTCC CACTACCGCT GGAAGGCCAG CCGGGCCGTG AGCGCGGTGG

           451                                              500
 Pv-C      GAACGGGCTT CCCTAAAAGC CAGCCCCGGG CCGGGGTCGG CATAGAATGA
 Pv-J      GAACGGGCTT CCCTAAAAGC CAGCCCCGGG CCGGGGTCGG CATAGAATGA
Consensus  GAACGGGCTT CCCTAAAAGC CAGCCCCGGG CCGGGGTCGG CATAGAATGA

           501                                              550
 Pv-C      AGGGGACGGC CCTAATGTTG TTGTGTTGGC AAAGCCAACT TCTTGGGTTG
 Pv-J      AGGGGACGGC CCTAATGTTG TTGTGTTGGC AAAGCCAACT TCTTGGGTTG
Consensus  AGGGGACGGC CCTAATGTTG TTGTGTTGGC AAAGCCAACT TCTTGGGTTG
           551                                              600
 Pv-C      CGGGCGGAGA AGAGCGGACG TGGGGACTCA GACCGGGGCG CAGCGTAACT
 Pv-J      CGGGCGGAGA AGAGCGGACG TGGGGACTCA GACCGGGGCG CAGCGTAACT
Consensus  CGGGCGGAGA AGAGCGGACG TGGGGACTCA GACCGGGGCG CAGCGTAACT
```

```
           601                                                 650
 Pv-C      AAGAGAGCCA TTCCATTTAG GGCGAGACAG AAGACAGGAC GCACTCTTTA
 Pv-J      AAGAGAGCCA TTCCATTTAG GGCGAGACAG AAGACAGGAC GCACTCTTTA
Consensus  AAGAGAGCCA TTCCATTTAG GGCGAGACAG AAGACAGGAC GCACTCTTTA

           651                                                 700
 Pv-C      CGTAGATAGA AGGCATTTCT CTATAAAAGT CTATCTCTAT CGAAAGAGTG
 Pv-J      CGTAGATAGA AGGCATTTCT CTATAAAAGT CTATCTCTAT CGAAAGAGTG
Consensus  CGTAGATAGA AGGCATTTCT CTATAAAAGT CTATCTCTAT CGAAAGAGTG

           701                                                 750
 Pv-C      AACCGCTAAC TGTGTGGTAA ATGGAGTAGG AGAGCCTTCC TAGGATCTGT
 Pv-J      AACCGCTAAC TGTGTGGTAA ATGGAGTAGG AGAGCCTTCC TAGGATCTGT
Consensus  AACCGCTAAC TGTGTGGTAA ATGGAGTAGG AGAGCCTTCC TAGGATCTGT

           751                                                 800
 Pv-C      TTTCATTGTG GCAATTGGGG CCTGGTAGAC TATTGCAGAC CTTGTTCGTC
 Pv-J      TTTCATTGTG GCAATTGGGG CCTGGTAGAC TATTGCAGAC CTTGTTCGTC
Consensus  TTTCATTGTG GCAATTGGGG CCTGGTAGAC TATTGCAGAC CTTGTTCGTC

           801                                                 850
 Pv-C      AGTATGAGCT TATGCTTAAC CTGCACGACA TGAAAGAGCA ACCTGGACAG
 Pv-J      AGTATGAGCT TATGCTTAAC CTGCACGACA TGAAAGAGCA ACCTGGACAG
Consensus  AGTATGAGCT TATGCTTAAC CTGCACGACA TGAAAGAGCA ACCTGGACAG

           851                                                 900
 Pv-C      TCTATTCTAA ACTTATATTG TTGTCCTATG TATGTTTTTT GGAGATTATT
 Pv-J      TCTATTCTAA ACTTATATTG TTGTCCTATG TATGTTTTTT GGAGATTATT
Consensus  TCTATTCTAA ACTTATATTG TTGTCCTATG TATGTTTTTT GGAGATTATT

           901                                                 950
 Pv-C      CCGCTCATGC TAAGAAGTTT CTTGACTCTC GCTCTCACTG ATGATTTTGA
 Pv-J      CCGCTCATGC TAAGAAGTTT CTTGACTCTC GCTCTCACTG ATGATTTTGA
Consensus  CCGCTCATGC TAAGAAGTTT CTTGACTCTC GCTCTCACTG ATGATTTTGA

           951                                                1000
 Pv-C      GCCTGCTCGA GCCTATTTAC TCCATCGCAG CCCCTTGCCT ACACTGTATA
 Pv-J      GCCTGCTCGA GCCTATTTAC TCCATCGCAG CCCCTTGCCT ACACTGTATA
Consensus  GCCTGCTCGA GCCTATTTAC TCCATCGCAG CCCCTTGCCT ACACTGTATA

           1001                                               1050
 Pv-C      GTGCTGTTGC AGAACTAATC TCTTCAGAGA CTTAACTTGG AACTATGAAA
 Pv-J      GTGCTGTTGC AGAACTAATC TCTTCAGAGA CTTAACTTGG AACTATGAAA
Consensus  GTGCTGTTGC AGAACTAATC TCTTCAGAGA CTTAACTTGG AACTATGAAA

           1051                                               1100
 Pv-C      TGACAATGAA CTTCATTAGT GAGGGCTACT TCTTCTATTA TACCTTCTTC
 Pv-J      TGACAATGAA CTTCATTAGT GAGGGCTACT TCTTCTATTA TACCTTCTTC
Consensus  TGACAATGAA CTTCATTAGT GAGGGCTACT TCTTCTATTA TACCTTCTTC

           1101                                               1150
 Pv-C      TGGCTAATCG AATTCCTGTT ACTGAAAGAA GCCTGGGAAT GGAATTCGAG
 Pv-J      TGGCTAATCG AATTCCTGTT ACTGAAAGAA GCCTGGGAAT GGAATTCGAG
Consensus  TGGCTAATCG AATTCCTGTT ACTGAAAGAA GCCTGGGAAT GGAATTCGAG

           1151                                               1200
 Pv-C      ACTGTCTCAC TCGGCCTCCT CGCAAGAACA CACCTAAAGG TAGTCAAACC
 Pv-J      ACTGTCTCAC TCGGCCTCCT CGCAAGAACA CACCTAAAGG TAGTCAAACC
Consensus  ACTGTCTCAC TCGGCCTCCT CGCAAGAACA CACCTAAAGG TAGTCAAACC
```

```
           1201                                                  1250
  Pv-C     CAGTCTAAAC CTCAAACTCA TTCCAAGGAA GGCTGCTGCT ACTAATGAGG
  Pv-J     CAGTCTAAAC CTCAAACTCA TTCCAAGGAA GGCTGCTGCT ACTAATGAGG
Consensus  CAGTCTAAAC CTCAAACTCA TTCCAAGGAA GGCTGCTGCT ACTAATGAGG

           1251                                                  1300
  Pv-C     ATTCTTCTTC TGAGCCTT.. ..TGAGTTAG TGAAAAGCCC TTCTCGTCAT
  Pv-J     ATTCTTCTTC TGAGCCTTCC TTTGAGTTAG TGAAAAGCCC TTCTCGTCAT
Consensus  ATTCTTCTTC TGAGCCTT.. ..TGAGTTAG TGAAAAGCCC TTCTCGTCAT

           1301                                                  1350
  Pv-C     AAGCTGCTCC TTCTCTAAGA CATTTTGCAT TTGTCCATCT CTAGCCTTTG
  Pv-J     AAGCTGCTCC TTCTCTAAGA CATTTTGCAT TTGTCCATCT CTAGCCTTTG
Consensus  AAGCTGCTCC TTCTCTAAGA CATTTTGCAT TTGTCCATCT CTAGCCTTTG

           1351                                                  1400
  Pv-C     AAAGCTGTCC TAATCCTCTG GTCAAGCTGG GCCAGGCTAG CTCTCTTATT
  Pv-J     AAAGCTGTCC TAATCCTCTG GTCAAGCTGG GCCAGGCTAG CTCTCTTATT
Consensus  AAAGCTGTCC TAATCCTCTG GTCAAGCTGG GCCAGGCTAG CTCTCTTATT

           1401                                                  1450
  Pv-C     CTGGATTGGC CATTATTAGA AGAACGAAGC CGTTAGGAGA GGCAGATCAG
  Pv-J     CTGGATTGGC CATTATTAGA AGAACGAAGC CGTTAGGAGA GGCAGATCAG
Consensus  CTGGATTGGC CATTATTAGA AGAACGAAGC CGTTAGGAGA GGCAGATCAG

           1451                                                  1500
  Pv-C     TCAAAGTATC TTGCTAGAAA GTCACTGCTA CCTTATTCAG TCTATCCCGG
  Pv-J     TCAAAGTATC TTGCTAGAAA GTCACTGCTA CCTTATTCAG TCTATCCCGG
Consensus  TCAAAGTATC TTGCTAGAAA GTCACTGCTA CCTTATTCAG TCTATCCCGG

           1501                                                  1550
  Pv-C     CAGTGCCTGA ATGGGAGTCT AGGCTAGCCA GTGCATCAAA CTTTTGACTG
  Pv-J     CAGTGCCTGA ATGGGAGTCT AGGCTAGCCA GTGCATCAAA CTTTTGACTG
Consensus  CAGTGCCTGA ATGGGAGTCT AGGCTAGCCA GTGCATCAAA CTTTTGACTG

           1551                                                  1600
  Pv-C     AAAGAACGAC TTTTCCAGCT AGATCGGACT AATTGTTTTT ATTACCGTAG
  Pv-J     AAAGAACGAC TTTTCCAGCT AGATCGGACT AATTGTTTTT ATTACCGTAG
Consensus  AAAGAACGAC TTTTCCAGCT AGATCGGACT AATTGTTTTT ATTACCGTAG

           1601                                                  1650
  Pv-C     GCATAATGAT AGCACTCAGC GGTTCGGGAA CTCTGCCTAT CTCAATCCCG
  Pv-J     GCATAATGAT AGCACTCAGC GGTTCGGGAA CTCTGCCTAT CTCAATCCCG
Consensus  GCATAATGAT AGCACTCAGC GGTTCGGGAA CTCTGCCTAT CTCAATCCCG

           1651                                                  1700
  Pv-C     GGATGAGAGA AGTTCAACAG GCAAGGCCGG CTTTGCTTCA TATGTAGCGG
  Pv-J     GGATGAGAGA AGTTCAACAG GCAAGGCCGG CTTTGCTTCA TATGTAGCGG
Consensus  GGATGAGAGA AGTTCAACAG GCAAGGCCGG CTTTGCTTCA TATGTAGCGG

           1701                                                  1750
  Pv-C     ATCGGTATTC TGCTATTCTC TCTCGCTTGA TACCGGACAT AGAACCCTAT
  Pv-J     ATCGGTATTC TGCTATTCTC TCTCGCTTGA TACCGGACAT AGAACCCTAT
Consensus  ATCGGTATTC TGCTATTCTC TCTCGCTTGA TACCGGACAT AGAACCCTAT

           1751                                                  1800
  Pv-C     CTCGATCAAC CCAACTGTCT AACCTTTCTT TCACATCACA GCTATCCACT
  Pv-J     CTCGATCAAC CCAACTGTCT AACCTTTCTT TCACATCACA GCTATCCACT
Consensus  CTCGATCAAC CCAACTGTCT AACCTTTCTT TCACATCACA GCTATCCACT
```

```
           1801                                                   1850
 Pv-C      CTCCTTTCTT TTTTTGAAAG TCAATCTTTT TTCGACGCTG GGCTTTAGTC
 Pv-J      CTCCTTTATT TTTTTGAAAG TCAATCTTTT TTCGACGCTG GGCTTTAGTC
Consensus  CTCCTTTaTT TTTTTGAAAG TCAATCTTTT TTCGACGCTG GGCTTTAGTC

           1851                                                   1900
 Pv-C      ATCCTAGTTC TACACAGGAA GGGAAGATAA TTACCAACTT CCATTGTCAA
 Pv-J      ATCCTAGTTC TACACAGGAA GGGAAGATAA TTACCAACTT CCATTGTCAA
Consensus  ATCCTAGTTC TACACAGGAA GGGAAGATAA TTACCAACTT CCATTGTCAA

           1901                                                   1950
 Pv-C      TCCCCAAACG GCTTGAAGCA TCTGGTATTG GTTCATCTAC CTTGATCTGC
 Pv-J      TCCCCAAACG GCTTGAAGCA TCTGGTATTG GTTCATCTAC CTTGATCTGC
Consensus  TCCCCAAACG GCTTGAAGCA TCTGGTATTG GTTCATCTAC CTTGATCTGC

           1951                                                   2000
 Pv-C      CTTCCGAGTT ACATGGAAGA CTGCCCCTTT CAAACTTCTT AGCTCAGAAA
 Pv-J      CTTCCGAGTT ACATGGAAGA CTGCCCCTTT CAAACTTCTT AGCTCAGAAA
Consensus  CTTCCGAGTT ACATGGAAGA CTGCCCCTTT CAAACTTCTT AGCTCAGAAA

           2001                                                   2050
 Pv-C      GATTGGAAGA TGAAAGCTAT CTCATTCAAC ATCGGACTTG TTGCTGTATT
 Pv-J      GATTGGAAGA TGAAAGCTAT CTCATTCAAC ATCGGACTTG TTGCTGTATT
Consensus  GATTGGAAGA TGAAAGCTAT CTCATTCAAC ATCGGACTTG TTGCTGTATT

           2051                                                   2100
 Pv-C      GATTCCCACT TGTTGAAGCT CTATCTGGTA CTCCCTTGCG CTAGAAGCTC
 Pv-J      GATTCCCACT TGTTGAAGCT CTATCTGGTA CTCCCTTGCG CTAGAAGCTC
Consensus  GATTCCCACT TGTTGAAGCT CTATCTGGTA CTCCCTTGCG CTAGAAGCTC

           2101                                                   2150
 Pv-C      TTGCTAGATT ATCCGACATT GACTCGCTAT AAGAAGATTG TCCGAATAGC
 Pv-J      TTGCTAGATT ATCCGACATT GACTCGCTAT AAGAAGATTG TCCGAATAGC
Consensus  TTGCTAGATT ATCCGACATT GACTCGCTAT AAGAAGATTG TCCGAATAGC

           2151                                                   2200
 Pv-C      ATAATTATTC TATATGTGAA AATAGTGATT GATAAGGGCT ATCAGATAGC
 Pv-J      ATAATTATTC TATATGTGAA AATAGTGATT GATAAGGGCT ATCAGATAGC
Consensus  ATAATTATTC TATATGTGAA AATAGTGATT GATAAGGGCT ATCAGATAGC

           2201                                                   2250
 Pv-C      AATGAGTGGA CTGGCAACCT GTACTTCTTT CTTTGACAGG CATTGCTGGT
 Pv-J      AATGAGTGGA CTGGCAACCT GTACTTCTTT CTTTGACAGG CATTGCTGGT
Consensus  AATGAGTGGA CTGGCAACCT GTACTTCTTT CTTTGACAGG CATTGCTGGT

           2251                                                   2300
 Pv-C      CCTAGGTCTG GTTGGGTAAA AAAGCTGGGG CATAGCTTCT ATTGCTGGCT
 Pv-J      CCTAGGTCTG GTTGGGTAAA AAAGCTGGGG CATAGCTTCT ATTGCTGGCT
Consensus  CCTAGGTCTG GTTGGGTAAA AAAGCTGGGG CATAGCTTCT ATTGCTGGCT

           2301                                                   2350
 Pv-C      TGTCTGCCCT GTCTCACTTA TAATTGAGGA CTTTTGCAGC GATATGTCGA
 Pv-J      TGTCTGCCCT GTCTCACTTA TAATTGAGGA CTTTTGCAGC GATATGTCGA
Consensus  TGTCTGCCCT GTCTCACTTA TAATTGAGGA CTTTTGCAGC GATATGTCGA

           2351                                                   2400
 Pv-C      TCAAAAAGTA ACTCCATTTT GACATGCCTA TCTTTGCTTT GAAAAACGAG
 Pv-J      TCAAAAAGTA ACTCCATTTT GACATGCCTA TCTTTGCTTT GAAAAACGAG
Consensus  TCAAAAAGTA ACTCCATTTT GACATGCCTA TCTTTGCTTT GAAAAACGAG
```

	2401				2450
Pv-C	TGGAGAGCTT	TTCCCCTATA	CGGAGATAGA	CTATCCTTAT	TAGATTAGGA
Pv-J	TGGAGAGCTT	TTCCCCTATA	CGGAGATAGA	CTATCCTTAT	TAGATTAGGA
Consensus	TGGAGAGCTT	TTCCCCTATA	CGGAGATAGA	CTATCCTTAT	TAGATTAGGA
	2451				2500
Pv-C	ATTTCCGTAA	CCGGTAGATT	GGATTTCCGG	TGGAAGTCAA	CAAGGACAGA
Pv-J	ATTTCCGTAA	CCGGTAGATT	GGATTTCCGG	TGGAAGTCAA	CAAGGACAGA
Consensus	ATTTCCGTAA	CCGGTAGATT	GGATTTCCGG	TGGAAGTCAA	CAAGGACAGA
	2501				2550
Pv-C	TTCCTGTTGG	CTAATCGAAT	GGAAATCCCA	TCTCATTTCC	GATTTGACTG
Pv-J	TTCCTGTTGG	CTAATCGAAT	GGAAATCCCA	TCTCATTTCC	GATTTGACTG
Consensus	TTCCTGTTGG	CTAATCGAAT	GGAAATCCCA	TCTCATTTCC	GATTTGACTG
	2551				2600
Pv-C	CCTAGACTGA	GCAAGCAATA	TCTTTGCTGT	CTCCTAGTGG	AATTAGCAGA
Pv-J	CCTAGACTGA	GCAAGCAATA	TCTTTGCTGT	CTCCTAGTGG	AATTAGCAGA
Consensus	CCTAGACTGA	GCAAGCAATA	TCTTTGCTGT	CTCCTAGTGG	AATTAGCAGA
	2601				2650
Pv-C	CTCCGACATG	AACTCTGAAA	CTCTGATTGC	TGTTTGAAAG	GGTTTACATA
Pv-J	CTCCGACATG	AACTCTGAAA	CTCTGATTGC	TGTTTGAAAG	GGTTTACATA
Consensus	CTCCGACATG	AACTCTGAAA	CTCTGATTGC	TGTTTGAAAG	GGTTTACATA
	2651				2700
Pv-C	GATTGCTATG	CTAATCCGTT	ATCTGATCAA	GACAAAAAAA	AAGGGATTGA
Pv-J	GATTGCTATG	CTAATCCGTT	ATCTGATCAA	GACAAAAAAA	AAGGGATTGA
Consensus	GATTGCTATG	CTAATCCGTT	ATCTGATCAA	GACAAAAAAA	AAGGGATTGA
	2701				2750
Pv-C	GATCAGTCTG	TCAATAGCAT	CAATGAGAGA	AGAAAGGACA	CTCTCCATCA
Pv-J	GATCAGTCTG	TCAATAGCAT	CAATGAGAGA	AGAAAGGACA	CTCTCCATCA
Consensus	GATCAGTCTG	TCAATAGCAT	CAATGAGAGA	AGAAAGGACA	CTCTCCATCA
	2751				2800
Pv-C	TGCAATGAGA	GAAGAGAGAT	GTACTGAAAG	CATGATCTCT	CTTACTTCTC
Pv-J	TGCAATGAGA	GAAGAGAGAT	GTACTGAAAG	CATGATCTCT	CTTACTTCTC
Consensus	TGCAATGAGA	GAAGAGAGAT	GTACTGAAAG	CATGATCTCT	CTTACTTCTC
	2801				2850
Pv-C	CAATAGAGAA	GGATAACCAA	ATCCTTCTCA	ATTGTTTCAC	TGCAGATAGC
Pv-J	CAATAGAGAA	GGATAACCAA	ATCCTTCTCA	ATTGTTTCAC	TGCAGATAGC
Consensus	CAATAGAGAA	GGATAACCAA	ATCCTTCTCA	ATTGTTTCAC	TGCAGATAGC
	2851				2900
Pv-C	ATCCAAAACT	CGGAAATGTA	ACAAAAGTCA	GATGCGGAAC	ACATGCAAGA
Pv-J	ATCCAAAACT	CGGAAATGTA	ACAAAAGTCA	GATGCGGAAC	ACATGCAAGA
Consensus	ATCCAAAACT	CGGAAATGTA	ACAAAAGTCA	GATGCGGAAC	ACATGCAAGA
	2901				2950
Pv-C	TTGCACTAGT	CACAGAGAGA	AGACAGCCAA	TCCAATAGAG	CAAGCAACCA
Pv-J	TTGCACTAGT	CACAGAGAGA	AGACAGCCAA	TCCAATAGAG	CAAGCAACCA
Consensus	TTGCACTAGT	CACAGAGAGA	AGACAGCCAA	TCCAATAGAG	CAAGCAACCA
	2951				3000
Pv-C	GTCACAGCAA	GGAAAGCAAA	GAGAGAAGCT	AGTCACAGGT	AAACATGCAC
Pv-J	GTCACAGCAA	GGAAAGCAAA	GAGAGAAGCT	AGTCACAGGT	AAACATGCAC
Consensus	GTCACAGCAA	GGAAAGCAAA	GAGAGAAGCT	AGTCACAGGT	AAACATGCAC

```
          3001                                               3050
  Pv-C    TGTCTCTAAG CTCGATTGAC TGGCCTAGTT TCACTCGGAG ATAGAGACCA
  Pv-J    TGTCTCTAAG CTCGATTGAC TGGCCTAGTT TCACTCGGAG ATAGAGACCA
Consensus TGTCTCTAAG CTCGATTGAC TGGCCTAGTT TCACTCGGAG ATAGAGACCA

          3051                                               3100
  Pv-C    GTTACCGAGC CAGTCAGATG CTTGCCTGGC CTTGTCTAAC TCCCCAGTTT
  Pv-J    GTTACCGAGC CAGTCAGATG CTTGCCTGGC CTTGTCTAAC TCCCCAGTTT
Consensus GTTACCGAGC CAGTCAGATG CTTGCCTGGC CTTGTCTAAC TCCCCAGTTT

          3101                                               3150
  Pv-C    CACAAGCAAG CCAGGCAGAT CACCTTGTTC AAGGATTTCA GAGCGAGTAC
  Pv-J    CACAAGCAAG CCAGGCAGAT CACCTTGTTC AAGGATTTCA GAGCGAGTAC
Consensus CACAAGCAAG CCAGGCAGAT CACCTTGTTC AAGGATTTCA GAGCGAGTAC

          3151                                               3200
  Pv-C    TTCCAACAAA ACGTCTGCCT AGACAGAGCT TCCAACCACT TGGACCCGAT
  Pv-J    TTCCAACAAA ACGTCTGCCT AGACAGAGCT TCCAACCACT TGGACCCGAT
Consensus TTCCAACAAA ACGTCTGCCT AGACAGAGCT TCCAACCACT TGGACCCGAT

          3201                                               3250
  Pv-C    GATACACCTG CAGGTCATCC TCTGTGAATC TGCAAGACAG GGAGAGAAAG
  Pv-J    GATACACCTG CAGGTCATCC TCTGTGAATC TGCAAGACAG GGAGAGAAAG
Consensus GATACACCTG CAGGTCATCC TCTGTGAATC TGCAAGACAG GGAGAGAAAG

          3251                                               3300
  Pv-C    ACATGCCGGA CTCACAAGAG AAAGACAGCC AATGCAGTCA GTCAGAGAGA
  Pv-J    ACATGCCGGA CTCACAAGAG AAAGACAGCC AATGCAGTCA GTCAGAGAGA
Consensus ACATGCCGGA CTCACAAGAG AAAGACAGCC AATGCAGTCA GTCAGAGAGA


          3301                                               3350
  Pv-C    GATGGTAATT CTGCTAACTG AATTCGGAAG ACTTGCTAGT CTAAGAGAGA
  Pv-J    GATGGTAATT CTGCTAACTG AATTCGGAAG ACTTGCTAGT CTAAGAGAGA
Consensus GATGGTAATT CTGCTAACTG AATTCGGAAG ACTTGCTAGT CTAAGAGAGA

          3351                                               3400
  Pv-C    TTGGACTCAT GCTTCCGGGA ATCTCAGTAG TATACCAGGA ATGCCCTATT
  Pv-J    TTGGACTCAT GCTTCCGGGA ATCTCAGTAG TATACCAGGA ATGCCCTATT
Consensus TTGGACTCAT GCTTCCGGGA ATCTCAGTAG TATACCAGGA ATGCCCTATT

          3401                                               3450
  Pv-C    GAGCGTCATG CCTGAACTCA TGTTCCGGAG AGTGCCTGTC GGATCACTTA
  Pv-J    GAGCGTCATG CCTGAACTCA TGTTCCGGAG AGTGCCTGTC GGATCACTTA
Consensus GAGCGTCATG CCTGAACTCA TGTTCCGGAG AGTGCCTGTC GGATCACTTA

          3451                                               3500
  Pv-C    TGCCTATGCA AGGAGAGAGC ACCGCTAAGG GAAAGTTCAA TCATTGGGAA
  Pv-J    TGCCTATGCA AGGAGAGAGC ACCGCTAAGG GAAAGTTCAA TCATTGGGAA
Consensus TGCCTATGCA AGGAGAGAGC ACCGCTAAGG GAAAGTTCAA TCATTGGGAA

          3501                                               3550
  Pv-C    GGTCAGACAC AAGAAACCCG AACTGCCTGA AAGGTAACTA GCTTGTCGTC
  Pv-J    GGTCAGACAC AAGAAACCCG AACTGCCTGA AAGGTAACTA GCTTGTCGTC
Consensus GGTCAGACAC AAGAAACCCG AACTGCCTGA AAGGTAACTA GCTTGTCGTC

          3551                                               3600
  Pv-C    TTCTTTCCTC AACCAGTGAC AGGCCTCACT ATCTACTGGA TTTGGTCTAC
  Pv-J    TTCTTTCCTC AACCAGTGAC AGGCCTCACT ATCTACTGGA TTTGGTCTAC
Consensus TTCTTTCCTC AACCAGTGAC AGGCCTCACT ATCTACTGGA TTTGGTCTAC
```

	3601 3650
Pv-C	CGGGCAGCTA GATCGGACTG GTAGCATGCT GAGAGTCCTG TGAGGAAGGT
Pv-J	CGGGCAGCTA GATCGGACTG GTAGCATGCT GAGAGTCCTG TGAGGAAGGT
Consensus	CGGGCAGCTA GATCGGACTG GTAGCATGCT GAGAGTCCTG TGAGGAAGGT
	3651 3700
Pv-C	CTTCCAAGAG AATGGCTGTA CCGATTCGAG ATGTGACAGC GGGTCAGTCA
Pv-J	CTTCCAAGAG AATGGCTGTA CCGATTCGAG ATGTGACAGC GGGTCAGTCA
Consensus	CTTCCAAGAG AATGGCTGTA CCGATTCGAG ATGTGACAGC GGGTCAGTCA
	3701 3750
Pv-C	ATAAAGCTGC CAGTTCTATT CTACGAGCTG CTTCCAAAGG TGAGAAAGCC
Pv-J	ATAAAGCTGC CAGTTCTATT CTACGAGCTG CTTCCAAAGG TGAGAAAGCC
Consensus	ATAAAGCTGC CAGTTCTATT CTACGAGCTG CTTCCAAAGG TGAGAAAGCC
	3751 3800
Pv-C	TGTTTCTCTG AGCTAGGACA CATGCCCGGA AAAGAATCCG AGGTCTATGA
Pv-J	TGTTTCTCTG AGCTAGGACA CATGCCCGGA AAAGAATCCG AGGTCTATGA
Consensus	TGTTTCTCTG AGCTAGGACA CATGCCCGGA AAAGAATCCG AGGTCTATGA
	3801 3850
Pv-C	TGCCGAACCG ACATCCCTGT TTGGAATAGG ATTTTCTTGA AAGGGACCGG
Pv-J	TGCCGAACCG ACATCCCTGT TTGGAATAGG ATTTTCTTGA AAGGGACCGG
Consensus	TGCCGAACCG ACATCCCTGT TTGGAATAGG ATTTTCTTGA AAGGGACCGG
	3851 3900
Pv-C	CTAACCACTC GCTCTCTCAG TCGAGCAAAC CTTTCGCTTT CCTTTGACTT
Pv-J	CTAACCACTC GCTCTCTCAG TCGAGCAAAC CTTTCGCTTT CCTTTGACTT
Consensus	CTAACCACTC GCTCTCTCAG TCGAGCAAAC CTTTCGCTTT CCTTTGACTT
	3901 3950
Pv-C	GCGGATACTT GCGAATAAAT AAGGACGGAC TATAGTGCTA CTGCTAGAGG
Pv-J	GCGGATACTT GCGAATAAAT AAGGACGGAC TATAGTGCTA CTGCTAGAGG
Consensus	GCGGATACTT GCGAATAAAT AAGGACGGAC TATAGTGCTA CTGCTAGAGG
	3951 4000
Pv-C	GGACTCTGCT AGGCTTGCTA ATTCGATCCT TGTCCGATAC GGCAGCTGCT
Pv-J	GGACTCTGCT AGGCTTGCTA ATTCGATCCT TGTCCGATAC GGCAGCTGCT
Consensus	GGACTCTGCT AGGCTTGCTA ATTCGATCCT TGTCCGATAC GGCAGCTGCT
	4001 4050
Pv-C	GGGTCTCCCC ATCTCTCCTA AACTTCCCCC GGTCTTCGGC CCGAGCTGTA
Pv-J	GGGTCTCCCC ATCTCTCCTA AACTTCCCCC GGTCTTCGGC CCGAGCTGTA
Consensus	GGGTCTCCCC ATCTCTCCTA AACTTCCCCC GGTCTTCGGC CCGAGCTGTA
	4051 4100
Pv-C	TGAGGCAGAA ACTCGCCCCA CGTACGGTTT GGAGTCCGAG CCCCACCCCA
Pv-J	TGAGGCAGAA ACTCGCCCCA CGTACGGTTT GGAGTCCGAG CCCCACCCCA
Consensus	TGAGGCAGAA ACTCGCCCCA CGTACGGTTT GGAGTCCGAG CCCCACCCCA
	4101 4122
Pv-C	TAATGGTGCG GCTTAGGTCA AC
Pv-J	TAATGGTGCG GCTTAGGTCA AC
Consensus	TAATGGTGCG GCTTAGGTCA AC

Figure S1. *ccmFc*i1 sequence alignments. DNA sequences of *ccmFc*i1 PCR amplification products were aligned on the Multalin (Corpet, 1988) interface web server <http://multalin.toulouse.inra.fr/multalin/> accessed 8/26/2022. A) Alignments revealing polymorphisms between and within plant genera. The *ccmFc*i1 sequences of *Citrus maxima* (*Cm*), *Poncirus trifoliata* (*Pt*), *Solanum lycopersicum* (*Sl*), *Vaccinium corymbosum* (*Vc*), *Cynodon dactylon* (*Cd*), *Cenchrus americanus* (*Ca*), and *Cenchrus purpureus* (*Cp*) were aligned revealing multiple indel and SNP polymorphisms between genera and, at position 804-807, a single, four nucleotide indel between the *Cenchrus* species. *Citrus* congener species *C. japonica*, *C. medica*, *C. paradisi*, and *C. reticulata* sequences did not differ from that of *C. maxima* and were not included in the alignment. This was also the case for *Solanum pennellii* as compared to *Sl*, *Vaccinium virgatum* as compared to *Vc*, and *Cynodon transvaalensis* as compared to *Cd*. B) Alignment of Andean *Phaseolus vulgaris* (Calima, *Pv*-C) and Mesoamerican *P. vulgaris* (Jamapa, *Pv*-J) *ccmFc*i1 sequences. This alignment shares 632 5' nucleotides and 133 3' nucleotides with other species and includes a 3353 nucleotide insertion (positions 633-3989 in the *Phaseolus* alignment) between the shared 5' and 3' intron blocks. The two *Phaseolus* accessions were polymorphic within the insertion but not in the 5' and 3' segments shared with other species. SNP and indel polymorphisms that distinguish congener species are highlighted in yellow.
