# Supplementary material for: Plant mitochondrial introns as genetic markers - conservation and variation
Source: Front Plant Sci. 2023 Mar 20;14:1116851. doi: 10.3389/fpls.2023.1116851 (PMC10067590; doi:10.3389/fpls.2023.1116851)
Supplement: Supplementary file 3 [file Image_2.pdf]

```
           1                                                  50
       Cm  TGTGCGAAGA GTGCGTTATG ACCCGTGGCC GCCTGCCTG. ..........
      Cme  TGTGCGAAGA GTGCGTTATG ACCCGTGGCC GCCTGCCTG. ..........
       Cr  TGTGCGAAGA GTGCGTTATG ACCCGTGGCC GCCTGCCTG. ..........
       Pt  TGTGCGAAGA GTGCGTTATG ACCCGTGGCC GCCTGCCTG. ..........
       Sl  TGTGCGAAGA GTGCGTTATG ACCCGTGGCC GCCTGCCTG. ..........
       Vc  TGTGCGAAGA GTGCGTTATG ACCCGTGGCC GCCCGCCTG. ..........
       Vv  TGTGCGAAGA GTGCGTTATG ACCCGTGGCC GCCCGCCTG. ..........
     Pv-C  TGTGCGAAGA GTGCGTTATG ACCCGTGGCC GCCTGCCTG. ..........
       Cd  TGTGCGAAGA GTGCGTTATG AACCGTGGCC GCCTACCTGC TTTCTTTGGT
       Ct  TGTGCGAAGA GTGCGTTATG AACCGTGGCC GCCTACCTGC TTT....GGT
       Ca  TGTGCGAAGA GTGCGTTATG AACCGTGGCC GCCTACCTGC TTT....GGT
       Cp  TGTGCGAAGA GTGCGTTATG AACCGTGGCC GCCTACCTGC TTT....GGT
Consensus  TGTGCGAAGA GTGCGTTATG AcCCGTGGCC GCCtgCCTG. ..........

           51                                                100
       Cm  ..GTGGGGGC GGCTCCTCCG TTGTGGGTAA ACGGGAACCC CGACTCTACG
      Cme  ..GTGGGGGC GGCTCCTCCG TTGTGGGTAA ACGGGAACCC CGACTCTACG
       Cr  ..GTGGGGGC GGCTCCTCCG TTGTGGGTAA ACGGGAACCC CGACTCTACG
       Pt  ..GTGGGGGC GGCTCCTCCG TTGTGGGTAA ACGGGAACCC CGACTCTACG
       Sl  ..GTGGGGGC GGCTCCTCCG TTGTGGGTAA ACGGGAAACC CGACTCTACG
       Vc  ..GTGGGGGC GGCTCCTCCG TTGTGGGTAA ACGGGAAACC CGACTCTACG
       Vv  ..GTGGGGGC GGCTCCTCCG TTGTGGGTAA ACGGGAAACC CGACTCTACG
     Pv-C  ..GTCTGGTA GGGTCCTCCG TTGTGGGTAA ACGGGAAACC CGACTCTACG
       Cd  TGGTGGGGGC GGCTCCTCCG TTGTGGGTAA ACGGGAAACC CGACTCTACG
       Ct  TGGTGGGGGC GGCTCCTCCG TTGTGGGTAA ACGGGAAACC CGACTCTACG
       Ca  TGGTGGGGGC GGCTCCTCCG TTGTGGGTAA ACGGGAAACC CGACTCTACG
       Cp  TGGTGGGGGC GGCTCCTCCG TTGTGGGTAA ACGGGAAACC CGACTCTACG
Consensus  ..GTggGGgc GGcTCCTCCG TTGTGGGTAA ACGGGAAaCC CGACTCTACG

           101                                               150
       Cm  AACCCGAAGA AAGGCTGCAC AGCAGTAGTA AGGGCGTTAA GACCGGAGCT
      Cme  AACCCGAAGA AAGGCTGCAC AGCAGTAGTA AGGGCGTTAA GACCGGAGCT
       Cr  AACCCGAAGA AAGGCTGCAC AGCAGTAGTA AGGGCGTTAA GACCGGAGCT
       Pt  AACCCGAAGA AAGGCTGCAC AGCAGTAGTA AGGGCGTTAA GACCGGAGCT
       Sl  AACCCGAGGA AAGGCTGCAC AGCAGTAGTA GGGGCGTTAA GACCGGAGCT
       Vc  AACCCGAGGA AAGGCTGCAC AGCAGTAGTA GGGGCGTTAA GACCGGAGCT
       Vv  AACCCGAGGA AAGGCTGCAC AGCAGTAGTA GGGGCGTTAA GACCGGAGCT
     Pv-C  AACCCGAGGA AAGGCTGCAC AGCAGTAGTA GGGGCGTTAA GACCGGAGCT
       Cd  AACCCGAGGA AAGGCTGCAC AGCTTCCGTA GGGGCGTTAA GACCGGAGCT
       Ct  AACCCGAGGA AAGGCTGCAC AGCTTCCGTA GGGGCGTTAA GACCGGAGCT
       Ca  AACCCGAGGA AAGGCTGCAC AGCTTCCGTA GGGGCGTTAA GACCGGAGCT
       Cp  AACCCGAGGA AAGGCTGCAC AGCTTCCGTA GGGGCGTTAA GACCGGAGCT
Consensus  AACCCGAgGA AAGGCTGCAC AGCagtaGTA gGGGCGTTAA GACCGGAGCT

           151                                               200
       Cm  TTTTGTAGTG CTAGCAGGAA TGCAAGTGAA TGAATCCCAT CCCCTAGCGA
      Cme  TTTTGTAGTG CTAGCAGGAA TGCAAGTGAA TGAATCCCAT CCCCTAGCGA
       Cr  TTTTGTAGTG CTAGCAGGAA TGCAAGTGAA TGAATCCCAT CCCCTAGCGA
       Pt  TTTTGTAGTG CTAGCAGGAA TGCAAGTGAA TGAATCCCAT CCCCTAGCGA
       Sl  TTTTGTAGTG CTAGCAGGAA TGCAAGTGAA TGAATCCCAT CCCCTAGCGA
       Vc  TTTTGTAGTG CTAGCAGGAA TGCAAGTGAA TGAATCCCAT CCCCTAGTGA
       Vv  TTTTGTAGTG CTAGCAGGAA TGCAAGTGAA TGAATCCCAT CCCCTAGTGA
     Pv-C  TTTTGTAGTG CTAGCAGGAA TGCAAGTGAA TGAATCCCAT CCCCTAGCGA
       Cd  TTTTGTAGTG CTAGCAGGAA TGCAAGTGAA TGAATCCAAT CCCAT.....
       Ct  TTTTGTAGTG CTAGCAGGAA TGCAAGTGAA TGAATCCAAT CCCAT.....
       Ca  TTTTGTAGTG CTAGCAGGAA TGCAAGTGAA TGAATCCAAT CCCAT.....
       Cp  TTTTGTAGTG CTAGCAGGAA TGCAAGTGAA TGAATCCAAT CCCAT.....
Consensus  TTTTGTAGTG CTAGCAGGAA TGCAAGTGAA TGAATCCcAT CCCcTag.ga
```

```
           201                                                250
      Cm   GTGAAGTGCT TACGCCCCTT TAGAGATAGG GGGGAGCAAA AGA....GCT
     Cme   GTGAAGTGCT TACGCCCCTT TAGAGATAGG GGGGAGCAAA AGA....GCT
      Cr   GTGAAGTGCT TACGCCCCTT TAGAGATAGG GGGGAGCAAA AGA....GCT
      Pt   GTGAAGTGCT TACGCCCCTT TAGAGATAGG GGGGAGCAAA AGA....GCT
      Sl   GTGAAGTGCT TACGCCCCTT TAGAGATAGG GGCGAGCAAA AGA....GCT
      Vc   GTGAAGTGCT TACGCCCCTG ACGAGATAGG GGCGAGCAAA AGAAAGAGCT
      Vv   GTGAAGTGCT TACGCCCCTG ACGAGATAGG GGCGAGCAAA AGAAAGAGCT
    Pv-C   GTGAAGTGCT TACGCCCCTT TA........ .......... ..........
      Cd   .......... ....CCCCT. ......C... GGCGAG.... ..........
      Ct   .......... ....CCCCT. ......C... GGCGAG.... ..........
      Ca   .......... ....CCCCTT TCAAATC... GGCGAG.... ..........
      Cp   .......... ....CCCCTT TCAAATC... GGCGAG.... ..........
Consensus  gtgaagtgct tacgCCCCTt t..a...... ggcgag.... ..........

           251                                                300
      Cm   CGTTCTTCCA TTTTGAGATC TTTAGATTCG TAAGATCATG ATAGAGTCCC
     Cme   CGTTCTTCCA TTTTGAGATC TTTAGATTCG TAAGATCATG ATAGAGTCCC
      Cr   CGTTCTTCCA TTTTGAGATC TTTAGATTCG TAAGATCATG ATAGAGTCCC
      Pt   CGTTCTTCCA TTTTGAGATC TTTAGATTCG TAAGATCATG ATAGAGTCCC
      Sl   CGTTCTTCCA TTTTGAGATC TTTAGATTCG TAAGATCCTA ATAGAGTCCC
      Vc   CGTTCTTCAA TTTGGAGATT TTTAGATTCG TAAGATCATG ATAGAGTCCC
      Vv   CGTTCTTCAA TTTGGAGATT TTTAGATTCG TAAGATCATG ATAGAGTCCC
    Pv-C   ...TATTCTA TT...AGA.. .......... .........G AAAGGGGC..
      Cd   .GTAATACCT TATTGA.... .......... .........A GTAGGGCA..
      Ct   .GTAATACCT TATTGA.... .......... .........A GTAGGGCA..
      Ca   .GTAATACCT TATTGA.... .......... .........A GTAGGGCA..
      Cp   .GTAATACCT TATTGA.... .......... .........A GTAGGGCA..
Consensus  .gttaTtCca TtttgAga.. .......... .........a atAGgG.c..

           301                                                350
      Cm   CTTTACCTTG AGATTCTATT AGTAAAGCGC TAGCCGCCCT ACAACTAGTA
     Cme   CTTTACCTTG AGATTCTATT AGTAAAGCGC TAGCCGCCCT ACAACTAGTA
      Cr   CTTTACCTTG AGATTCTATT AGTAAAGCGC TAGCCGCCCT ACAACTAGTA
      Pt   CTTTACCTTG AGATTCTATT AGTAAAGCGC TAGCCGCCCT ACAACTAGTA
      Sl   TTT.ACTTT. CTATTATATT AGTCAAGCGC TAGC.GCCCT ACAACTAGTA
      Vc   CTA.ACCTTG ATATTCTATT AGTCAAGCGC TAGC.GCCCT AGAACTAGTA
      Vv   CTA.ACCTTG ATATTCTATT AGTCAAGCGC TAGC.GCCCT AGAACTAGTA
    Pv-C   .......... .......... ......GCGC TAGC...... ..........
      Cd   .......... .......... .......CGC TA........ ..........
      Ct   .......... .......... .......CGC TA........ ..........
      Ca   .......... .......... .......CGC TA........ ..........
      Cp   .......... .......... .......CGC TA........ ..........
Consensus  .......... .......... ......gCGC TAgc...... ..........

           351                                                400
      Cm   TAGTATAGCA GATCAGCCAG CTTCAAAACT TACCTTATTA TTAGAGAAAG
     Cme   TAGTATAGCA GATCAGCCAG CTTCAAAACT TACCTTATTA TTAGAGAAAG
      Cr   TAGTATAGCA GATCAGCCAG CTTCAAAACT TACCTTATTA TTAGAGAAAG
      Pt   TAGTATAGCA GATCAGCCAG CTTCAAAACT TACCTTATTA TTAGAGAAAG
      Sl   TAGTATAGCA GAGCAGCCAG CTTCAAAGCT TACCTGATTC AAAAAAAAAA
      Vc   TAGTATAGCA GAGCAGCCAG CTTCAAAGCT TACCTTATTA TTAGAGAAAG
      Vv   TAGTATAGCA GAGCAGCCAG CTTCAAAGCT TACCTTATTA TTAGAGAAAG
    Pv-C   .......... .........G CTTCAA.... .....TAATA ATAGAAA...
      Cd   .......... .......CGG CAACACAG.. .......... ..........
      Ct   .......... .......CGG CAACACAG.. .......... ..........
      Ca   .......... .......CGG CAACACAG.. .......... ..........
      Cp   .......... .......CGG CAACACAG.. .......... ..........
Consensus  .......... .......c.G CttCAaag.. ......a.t. ..a.a.a...
```

```
          401                                                450
       Cm GG........ .......... .......... ...GGCTGAG AAGCAGTT..
      Cme GG........ .......... .......... ...GGCTGAG AAGCAGTT..
       Cr GG........ .......... .......... ...GGCTGAG AAGCAGTT..
       Pt GG........ .......... .......... ...GGCTGAG AAGCAGTT..
       Sl GAA.GTATT. ........CT AAAAAAAGAG AAAGGTTAGG GGGCTGAGAA
       Vc GGGCGCGCTA GCGCAGGGCT TTACTAATAG AAAGGTTAGG AGGCTGAGAA
       Vv GGGCGCGCTA GCGCAGGGCT TTACTAATAG AAAGGTTAGG AGGCTGAGAA
     Pv-C .......... .......... .......... ...GGTTAGG GGGCTGAGAA
       Cd .......... .......... .......... ...GACTGAC GGGGTGGGG.
       Ct .......... .......... .......... ...GACTGAC GGGGTGGGG.
       Ca .......... .......... .......... ...GACTGAC GGGGTGGGG.
       Cp .......... .......... .......... ...GACTGAC GGGGTGGGG.
Consensus .......... .......... .......... ...GgcTgag ggGctG.g..

          451                                                500
       Cm .......... ...GATTGCT GTCTACTCCG CGAGCCTTCA CTGTCCCGTT
      Cme .......... ...GATTGCT GTCTACTCCG CGAGCCTTCA CTGTCCCGTT
       Cr .......... ...GATTGCT GTCTACTCCG CGAGCCTTCA CTGTCCCGTT
       Pt .......... ...GATTGCT GTCTACTCCG CGAGCCTTCA CTGTCCCGTT
       Sl GCTCTTGCTT GCTGCTTGCT GTCTACTTCG CGAGCCTTCA CTGCCCCGTT
       Vc G......... ...GCTTGCT GTCTACTTCG CGAGCCTTCA CTGCCCCGTT
       Vv G......... ...GCTTGCT GTCTACTTCG CGAGCCTTCA CTGCCCCGTT
     Pv-C AA........ ...GCTTGCT GTCTACTTCT CGAGCCTTCA CTGCCCCGTT
       Cd .......... ...GCTTGCT GTCTACTTGG CGAGCCTTCA CTGCCCCCTT
       Ct .......... ...GCTTGCT GTCTACTTGG CGAGCCTTCA CTGCCCCCTT
       Ca .......... ...GCTTGCT GTCTACTTGG CGAGCCTTCA CTGCCCCCTT
       Cp .......... ...GCTTGCT GTCTACTTGG CGAGCCTTCA CTGCCCCCTT
Consensus .......... ...GcTTGCT GTCTACTtcg CGAGCCTTCA CTGcCCCgTT

          501                                                550
       Cm CTGTAACTTC CCTTCTTTCG TCCGTCCACG AGGCTGTAAA AAGA....GA
      Cme CTGTAACTTC CCTTCTTTCG TCCGTCCACG AGGCTGTAAA AAGA....GA
       Cr CTGTAACTTC CCTTCTTTCG TCCGTCCACG AGGCTGTAAA AAGA....GA
       Pt CTGTAACTTC CCTTCTTTCG TCCGTCCACG AGGCTGTAAA AAGA....GA
       Sl CTGTAACTTC CCTTCTTTCG TCCGTCCACG AGGCTGTAAA AAGA....GA
       Vc CTGTAACTTC CCTTCTTTCG TCCGTCCACG AGGCTGTAAA AAGA....GA
       Vv CTGTAACTTC CCTTCTTTCG TCCGTCCACG AGGCTGTAAA AAGA....GA
     Pv-C CTGTAACTTC CCTTCTTTAG TCCGTCCACG AGGCTGTAAA AATA....TA
       Cd CTGTAACTTC CCTTCTTTCG TCCGTCCACG AGGCAGTCAA AAGAAAGAGA
       Ct CTGTAACTTC CCTTCTTTCG TCCGTCCACG AGGCAGTCAA AAGAAAGAGA
       Ca CTGTAACTTC CCTTCTTTCG TCCGTCCACG AGGCAGTCAA AAGAAAGAGA
       Cp CTGTAACTTC CCTTCTTTCG TCCGTCCACG AGGCAGTCAA AAGAAAGAGA
Consensus CTGTAACTTC CCTTCTTTcG TCCGTCCACG AGGCtGTaAA AAgA....gA

          551                                                600
       Cm GAAAGGGGCG GCTATCTAGC GGGAGTCGTT TCGGTTGTAT GCCACGAGGT
      Cme GAAAGGGGCG GCTATCTAGC GGGAGTCGTT TCGGTTGTAT GCCACGAGGT
       Cr GAAAGGGGCG GCTATCTAGC GGGAGTCGTT TCGGTTGTAT GCCACGAGGT
       Pt GAAAGGGGCG GCTATCTAGC GGGAGTCGTT TCGGTTGTAT GCCACGAGGT
       Sl GAAAGGGGCG GCTATCTAGC GGGAGTCGTT TCGGTTGTAT GCCACGAGGT
       Vc GAAAGGGGCG GCTATCTAGC GGGAGTCGTT TCGGTTGTAT GCCACGAGGT
       Vv GAAAGGGGCG GCTATCTAGC GGGAGTCGTT TCGGTTGTAT GCCACGAGGT
     Pv-C GATAGGGGCG GCTATCTAGC GGGAGTCGTT TCGGTTGTAT GCCACGAGGT
       Cd GAAAGGGGCG GCTATAACGC AGGAGTCGTG GCGGTTGTAT GCCACAAGGT
       Ct GAAAGGGGCG GCTATAACGC AGGAGTCGTG GCGGTTGTAT GCCACAAGGT
       Ca GAAAGGGGCG GCTATAACGC AGGAGTCGTG GCGGTTGTAT GCCACAAGGT
       Cp GAAAGGGGCG GCTATAACGC AGGAGTCGTG GCGGTTGTAT GCCACAAGGT
Consensus GAaAGGGGCG GCTATctaGC gGGAGTCGTt tCGGTTGTAT GCCACgAGGT
```

```
            601                                                650
       Cm   CCCTATGGAC AAGGGGACAA GTGAATCATC GCTTTTGGGC GCAGGCAGCC
      Cme   CCCTATGGAC AAGGGGACAA GTGAATCATC GCTTTTGGGC GCAGGCAGCC
       Cr   CCCTATGGAC AAGGGGACAA GTGAATCATC GCTTTTGGGC GCAGGCAGCC
       Pt   CCCTATGGAC AAGGGGACAA GTGAATCATC GCTTTTGGGC GCAGGCAGCC
       Sl   CCCTATGGAC AAGGGGACAA GTGAATCATC GCTTTTGGGC GCAGGCAGCC
       Vc   CCCTATGGAC AAGGGGACAA GTGAATCATC GCTTTTGGGC GCAGGCAGCC
       Vv   CCCTATGGAC AAGGGGACAA GTGAATCATC GCTTTTGGGC GCAGGCAGCC
     Pv-C   CCCTATGGAC AAGGGGA... ....ATCATC GCTTTTGGGC GCAGGCAGCC
       Cd   CCCTATAGAC AAGGGGACAA GTGAATCATC GCTTTTGGGC GCAGGCAGCC
       Ct   CCCTATAGAC AAGGGGACAA GTGAATCATC GCTTTTGGGC GCAGGCAGCC
       Ca   CCCTATAGAC AAGGGGACAA GTGAATCATC GCTTTTGGGC GCAGGCAGCC
       Cp   CCCTATAGAC AAGGGGACAA GTGAATCATC GCTTTTGGGC GCAGGCAGCC
Consensus   CCCTATgGAC AAGGGGAcaa gtgaATCATC GCTTTTGGGC GCAGGCAGCC

            651                                                700
       Cm   CTCTACCATC CATCCCATTG CATTCCATCG TCTCGTATTG TACTGTACCG
      Cme   CTCTACCATC CATCCCATTG CATTCCATCG TCTCGTATTG TACTGTACCG
       Cr   CTCTACCATC CATCCCATTG CATTCCATCG TCTCGTATTG TACTGTACCG
       Pt   CTCTACCATC CATCCCATTG CATTCCATCG TCTCGTATTG TACTGTACCG
       Sl   CTCTACCATC TATCCCATTG CATTCCATCG TCTCGTATTG TACTGTGCCG
       Vc   CTCTACCATC TATCCCATTG CATTCCATCG TCTCGTATTG TACTGTACCG
       Vv   CTCTACCATC TATCCCATTG CATTCCATCG TCTCGTATTG TACTGTACCG
     Pv-C   CTCTACCATC CATCCCATTG CATTCCATCG TCTCGTATTG TACTGTACCG
       Cd   CTCTACCAGT CTTCCCATTG CATTCC.... TCTCATAGAG TACTGTACCG
       Ct   CTCTACCATC CATCCCATTG CATTCC.... TCTCATAGAG TACTGTACCG
       Ca   CTCTACCATC CATCCCATTG CATTCC.... TCTCATAGAG TACTGTACCG
       Cp   CTCTACCATC CATCCCATTG CATTCC.... TCTCATAGAG TACTGTACCG
Consensus   CTCTACCAtc caTCCCATTG CATTCCatcg TCTCgTAttG TACTGTACCG

            701                                                750
       Cm   TA.....CCA GATACATCTA TTGAGTGAGA GAAAGGTA.. GTGTAACTAA
      Cme   TA.....CCA GATACATCTA TTGAGTGAGA GAAAGGTA.. GTGTAACTAA
       Cr   TA.....CCA GATACATCTA TTGAGTGAGA GAAAGGTA.. GTGTAACTAA
       Pt   TA.....CCA GATACATCTA TTGAGTGAGA GAAAGGTA.. GTGTAACTAA
       Sl   TATCAGACCA GATAGATCTA TTGAGTGAGA GAAAGGGA.. GAAAACTCTA
       Vc   TATCAGACCA GATAGATCTA TTGAGTGAGA GAAAGGAACG TAGTAATTAT
       Vv   TATCAGACCA GATAGATCTA TTGAGTGAGA GAAAGGAACG TAGTAATTAT
     Pv-C   TACCAAACCA GATACATCTA TTGAGTGAGA GAAAGGTAGC TTCTCTATAG
       Cd   TCTCAGACCA GATACATCTA TGGAAAGAAA GGAT...... .....TTCAT
       Ct   TCTCAGACCA GATACATCTA TGGAAAGAAA GGAT...... .....TTCAT
       Ca   TCTCAGACCA GATACATCTA TGGAAAGAAA GGAT...... .....TTCAT
       Cp   TCTCAGACCA GATACATCTA TGGAAAGAAA GGAT...... .....TTCAT
Consensus   TatcagaCCA GATAcATCTA TtGAgtGAgA GaAagg.a.. .....ttcAt

            751                                                800
       Cm   TTATA.TATT CATTCA.TTA TTAATAGGGA CCCCCATTCA TTCCTAGATT
      Cme   TTCTA.TATT CATTCA.TTA TTAATAGGGA CCCCCATTCA TTCCTAGATT
       Cr   TTATA.TATT CATTCA.TTA TTAATAGGGA CCCCCATTCA TTCCTAGATT
       Pt   TTATA.TATT CATTCA.TTA TTAATAGGGA CCCCCATTCA TTCCTAGATT
       Sl   ATTAGATATT CTTTTT.CTA TTGATAGGGA TCCCCATTCA TTCCTAGATT
       Vc   ATATATTCTT CATTTTGATA TTGATAGGGA TCCCCATTCA TTCCTAGATT
       Vv   ATATATTCTT CATTTTGATA TTGATAGGGA TCCCCATTCA TTCCTAGATT
     Pv-C   ATATATTATC AATTCA.TTT TTGATATGGA T........A GGGCCAGATT
       Cd   AGAAAATCTG CATTTTTCT. TTTTTTTGGA TCCC.ATGCA TTCCGTTATT
       Ct   AGAAAATCTG CATTTTTCT. TTTTTTTGGA TCCC.ATGCA TTCCGTTATT
       Ca   AGAAAATATT CTTTTTTCTA TTTTTTTGGA TCCCCATGCA TTCCGTTATT
       Cp   AGAAAATATT CATTTTTCTA TTTTTTTGGA TCCCCATGCA TTCCGTTATT
Consensus   ataaAaTaTt caTTtt.cTa TT.aTatGGA tccccat.cA ttcC.agATT
```

```
          801                                                850
       Cm CCCGTCACTA CGGATTGATT GTCCCTACCT AACTACATGG TAGTGGTCTA
      Cme CCCGTCACTA CGGATTGATT GTCCCTACCT AACTACATGG TAGTGGTCTA
       Cr CCCGTCACTA CGGATTGATT GTCCCTACCT AACTACATGG TAGTGGTCTA
       Pt CCCGTCACTA CGGATTGATT GTCCCTACCT AACTACATGG TAGTGGTCTA
       Sl CCCGTCACTA CGGACTTATT GTCCCTACCT AACTACATGG TAGTGGTCTA
       Vc CCCGTCACTA CGGATTGATT GTCCCTACCT AACTACATGG TAGTGGTCTA
       Vv CCCGTCACTA CGGATTGATT GTCCCTACCT AACTACATGG TAGTGGTCTA
     Pv-C CCCGTCACTA CGGATTGATT GTCTCTACCT AACTACATGG TAGTGGTCTA
       Cd CCCGTAACTA TGGATTGATT GTCCCTACCT AACTAAATGG TAGTGGTCTA
       Ct CCCGTAACTA TGGATTGATT GTCCCTACCT AACTAAATGG TAGTGGTCTA
       Ca CCCGTAACTA TGGATTGATT GTCCCTACCT AACTAAATGG TAGTGGTCTA
       Cp CCCGTAACTA TGGATTGATT GTCCCTACCT AACTAAATGG TAGTGGTCTA
Consensus CCCGTcACTA cGGATTGATT GTCcCTACCT AACTAcATGG TAGTGGTCTA

          851                                                900
       Cm GGGAGCGAAA TTGCTAGAGC ACGGGAGAAT GAAGAGTAAT GATTTCAGCA
      Cme GGGAGCGAAA TTGCTAGAGC ACGGGAGAAT GAAGAGTAAT GATTTCAGCA
       Cr GGGAGCTAAA TTGCTAGAGC ACGGGAGAAT GAAGAGTAAT GATTTCAGCA
       Pt GGGAGCTAAA TTGCTAGAGC ACGGGAGAAT GAAGAGTAAT GATTTCAGCA
       Sl GGGAGCGCAA TTGCTAGAGC ACGGGAGAAT TAAGAGTAAT GATTTCAGCA
       Vc GGGAGCGCAA TTGCTAGAGC ACGGGAGAAT GAAGAGTAAT GATTTCAGCA
       Vv GGGAGCGCAA TTGCTAGAGC ACGGGAGAAT GAAGAGTAAT GATTTCAGCA
     Pv-C GGGAGCGCAA TTGCTAGAGC ACGGGAGAAT TAAGAGTAAT GATTTCAGCA
       Cd GGGAGCACAA TTGCTAGAGC ACGGGAGAAT GAAGAGTAAT GATTTAAGCA
       Ct GGGAGCACAA TTGCTAGAGC ACGGGAGAAT GAAGAGTAAT GATTTAAGCA
       Ca GGGAGCGCAA TTGCTAGAGC ACGGGAGAAT GAAGAGTAAT GATTTAAGCA
       Cp GGGAGCGCAA TTGCTAGAGC ACGGGAGAAT GAAGAGTAAT GATTTAAGCA
Consensus GGGAGCgcAA TTGCTAGAGC ACGGGAGAAT gAAGAGTAAT GATTTcAGCA

          901                                                950
       Cm AGAGCAGCCG GACGGACTAC TATAGTGAGT CTAGTGACTA CTAGAGTAGA
      Cme AGAGCAGCCG GACGGACTAC TATAGTGAGT CTAGTGACTA CTAGAGTAGA
       Cr AGAGCAGCCG GACGGACTAC TATAGTGAGT CTAGTGACTA CTAGAGTAGA
       Pt AGAGCAGCCG GACGGACTAC TATAGTGAGT CTAGTGACTA CTAGAGTAGA
       Sl AGAGCAGCCA GACGGACTAC TATAGTGAGT CTAGTGACTA CTAGAGTAGA
       Vc AGAGCAGCCG GACGGACTAC TGACGTGAGT CTAGTGACTA CTAGAGTAGA
       Vv AGAGCAGCCG GACGGACTAC TGACGTGAGT CTAGTGACTA CTAGAGTAGA
     Pv-C AGAGCAGCCG GACGGACTAC TATAGTGAGT CCAGTGACTA CTAGAGTAGA
       Cd AGAGCAGCCG GACGGACTAC TATAGTCAGT CTAGTGACTA CTATAGTCTT
       Ct AGAGCAGCCG GACGGACTAC TATAGTCAGT CTAGTGACTA CTATAGTCTT
       Ca AGAGCAGCCG GACGGACTAC TATAGTCAGT CTAGTGACTA CTATAGTCTT
       Cp AGAGCAGCCG GACGGACTAC TATAGTCAGT CTAGTGACTA CTATAGTCTT
Consensus AGAGCAGCCG GACGGACTAC TataGTgAGT CtAGTGACTA CTAgAGTaga

          951                                               1000
       Cm GT........ .TGAGTCAAA A....GGTAT GGTATAGCGG CCTTTCCGAG
      Cme GT........ .TGAGTCAAA A....GGTAT GGTATAGCGG CCTTTCCGAG
       Cr GT........ .TGAGTCAAA A....GGTAT GGTATAGCGG CCTTTCCGAG
       Pt GT........ .TGAGTCAAA A....GGTAT GGTATAGCGG CCTTTCCGAG
       Sl GT........ .TAAGTCAAA G....GGTAT GGTATAGCAG CCTTTCCGAG
       Vc GT........ .TGAGTCAAA AAAAAGGTAT GGTATAGCAG CCTTTCCGAG
       Vv GT........ .TGAGTCAAA AAAAAGGTAT GTTATAGCAG CCTTTCCGAG
     Pv-C GT........ .GGAGTCAAA A....GGTAT GGTATAGCAG CCTTTCCGAG
       Cd TTCTTTCTTT GTAGTTAAGT AATAATGTTT GGTATAGCAG CCTTTCCGAG
       Ct TTCTTTCTTT GTAGTTAAGT AATAATGTTT GGTATAGCAG CCTTTCCGAG
       Ca TTCTTTCTTT GTAGTTAAGT AATAATGTTT GGTATAGCAG CCTTTCCGAG
       Cp TTCTTTCTTT GTAGTTAAGT AATAATGTTT GGTATAGCAG CCTTTCCGAG
Consensus gT........ .taagTcAaa Aa.aagGTaT GGTATAGCaG CCTTTCCGAG
```

```
           1001                                              1050
       Cm  CGAGCCTCCG GGATCTCCTG TAAACCCCCA TGATGTGGTA AAGGGAGGAT
      Cme  CGAGCCTCCG GGATCTCCTG TAAACCCCCA TGATGTGGTA AAGGGAGGAT
       Cr  CGAGCCTCCG GGATCTCCTG TAAACCCCCA TGATGTGGTA AAGGGAGGAT
       Pt  CGAGCCTCCG GGATCTCCTG TAAACCCCCA TGATGTGGTA AAGGGAGGAT
       Sl  CGAGCCTCTG GGATCTCCTG TAAACCCCCA TGATGTGGAA AAGGGAGGAT
       Vc  CGAGCCTCTG GGATCTCCTG TAAACCCCCA TGATGTGGTA AAGGGAGGAT
       Vv  CGAGCCTCTG GGATCTCCTG TAAACCCCCA TGATGTGGTA AAGGGAGGAT
     Pv-C  CGAGCCTCTG GGATCTCCTG TAAACCCCCA TGATGTGGTA AAGGTAGGAT
       Cd  CGAGTCTCTG GGATCTCCTG TAAACCCCCA TGATGTGGTA AAGGGAGGAT
       Ct  CGAGTCTCTG GGATCTCCTG TAAACCCCCA TGATGTGGTA AAGGGAGGAT
       Ca  CGAGTCTCTG GGATCTCCTG TAAACCCCCA TGATGTGGTA AAGGGAGGAT
       Cp  CGAGTCTCTG GGATCTCCTG TAAACCCCCA TGATGTGGTA AAGGGAGGAT
Consensus  CGAGcCTCtG GGATCTCCTG TAAACCCCCA TGATGTGGTA AAGGgAGGAT

           1051                                              1100
       Cm  ATTAGGGGAA GCAGTGAGTG GAGATTCCCC TGCGGAGAGC CGGATGAGGG
      Cme  ATTAGGGGAA GCAGTGAGTG GAGATTCCCC TGCGGAGAGC CGGATGAGGG
       Cr  ATTAGGGGAA GCAGTGAGTG GAGATTCCCC TGCGGAGAGC CGGATGAGGG
       Pt  ATTAGGGGAA GCAGTGAGTG GAGATTCCCC TGCGGAGAGC CGGATGAGGG
       Sl  ATTAGGGGAA GCAGTGAGTG GAGATTCCCC TGCAGAGAGC CGGATGAGGG
       Vc  ATTAGGGGAA GCAGTGAGTG GAGATTCCCC TGCAGAGAGC CGGATGAGGG
       Vv  ATTAGGGGAA GCAGTGAGTG GAGATTCCCC TGCAGAGAGC CGGATGAGGG
     Pv-C  ATTAGGGGAA GCAGTGAGTG GAGATTCCCC TGCAGAGAGC CGGATGAGGG
       Cd  ATTAGGGGAA GCAGTGAGTG GAGATTCCCC TGCAGAGAGC CGGATGAGGG
       Ct  ATTAGGGGAA GCAGTGAGTG GAGATTCCCC TGCAGAGAGC CGGATGAGGG
       Ca  ATTAGGGGAA GCAGTGAGTG GAGATTCCCC TGCAGAGAGC CGGATGAGGG
       Cp  ATTAGGGGAA GCAGTGAGTG GAGATTCCCC TGCAGAGAGC CGGATGAGGG
Consensus  ATTAGGGGAA GCAGTGAGTG GAGATTCCCC TGCaGAGAGC CGGATGAGGG

              1101                                        1146
       Cm  GAGACCTTCA CGTCCGGTTC GGAGGGCGGG GATATCCCGA CCCTAC
      Cme  GAGACCTTCA CGTCCGGTTC GGAGGGCGGG GATATCCCGA CCCTAC
       Cr  GAGACCTTCA CGTCCGGTTC GGAGGGCGGG GATATCCCGA CCCTAC
       Pt  GAGACCTTCA CGTCCGGTTC GGAGGGCGGG GATATCCCGA CCCTAC
       Sl  GAGACCCTCA CGTCCGGTTC GGAGGGCGGG GATATCCCGA CCCTAC
       Vc  GAGACCTTCA CGTCCGGTTC GGAGGGCGGG GATATCCCGA CCCTAC
       Vv  GAGACCTTCA CGTCCGGTTC GGAGGGCGGG GATATCCCGA CCCTAC
     Pv-C  GAGACCTTCA CGTCCGGTTC GGAGGGCGGG GATATCCCGA CCCTAC
       Cd  GAGACCCTCA CGTCCGGTTC GGAGGGCGGG GATATCCCGA CCCTAC
       Ct  GAGACCCTCA CGTCCGGTTC GGAGGGCGGG GATATCCCGA CCCTAC
       Ca  GAGACCCTCA CGTCCGGTTC GGAGGGCGGG GATACCCCGA CCCTAC
       Cp  GAGACCCTCA CGTCCGGTTC GGAGGGCGGG GATACCCCGA CCCTAC
Consensus  GAGACCcTCA CGTCCGGTTC GGAGGGCGGG GATAtCCCGA CCCTAC
```

Figure S2. *nad5*i4 sequence alignments. DNA sequences of *nad5*i4 PCR amplification products were aligned on the Multalin (Corpet, 1998) interface web server <http://multalin.toulouse.inra.fr/multalin/> accessed 8/26/2022. The *nad5*i4 sequences of *Citrus Citrus maxima (Cm)*, *Citrus reticulata* (*Cr*), *Citrus medica* (*Cme*), *Poncirus trifoliata* (*Pt*), *Solanum lycopersicum* (*Sl*), *Phaseolus vulgaris* 'Calima' (*Pv*-C), *Vaccinium corymbosum* (*Vc*), *Vaccinium virgatum* (*Vg*), *Cynodon dactylon* (*Cd*), *Cynodon transvaalensis* (*Ct*), *Cenchrus americanus* (*Ca*), and *Cenchrus purpureus* (*Cp*) were aligned revealing multiple indel and SNP polymorphisms between genera. *Citrus* species were distinguished by SNPs at positions 753 and 857, and *Vaccinium* species were distinguished by a SNP at position 982. *Cynodon* species were distinguished by an indel at positions 44-47 and SNPs at positions 659, 660 and 662. *Cenchrus* species were distinguished by a SNP at position 762. Entries that did not differ, *Citrus paradisi* as compared to *Cm, Citrus japonica* as compared to *Cr*, *Solanum pennellii* as compared to *Sl*, and *Phaseolus vulgaris* 'Jamapa' as compared to *Pv*-C, were not included in the alignment. SNP and indel polymorphisms that distinguished congener species are highlighted in yellow.
