# Supplementary material for: Plant mitochondrial introns as genetic markers - conservation and variation
Source: Front Plant Sci. 2023 Mar 20;14:1116851. doi: 10.3389/fpls.2023.1116851 (PMC10067590; doi:10.3389/fpls.2023.1116851)
Supplement: Supplementary file 4 [file Image_3.pdf]

```
           1                                                 50
       Cm  GTGCGGCACG AAGCCGCTGA CGCCGAGTCG GCTCCTATGC CGCTAGCTAT
       Cr  GTGCGGCACG AAGCCGCTGA CGCCGAGTCG GCTCCTATGC CGCTAGCTAT
      Cme  GTGCGGCACG AAGCCGCTGA CGCCGAGTCG GCTCCTATGC CGCTAGCTAT
       Pt  GTGCGGCACG AAGCCGCTGA CGCCGAGTCG GCTCCTATGC CGCTAGCTAT
       Sl  GTGCGGCACG AAGCCGCTGA TGCCGAGTCG GCTCCTATGC CGCTAGCTAT
     Pv-C  GTGCGGCACG AAGCCGCTGA CGCCGAGTCG GCTCCTATGC CGCTAGCTAT
       Vc  GTGCGGCACG AAGCCGCTGA CGCCGAGTCG GCTCCTATGC CGCTAGCTAT
       Cd  GTGCGGCACG AAGCCGCTGA CGCTGAGTAG GCTCCTATGC CGCTAGC...
       Ca  GTGCGGCACG AAGCCGCTGA CGCTGAGTAG GCTCCTATGC CGCTAGC...
       Cp  GTGCGGCACG AAGCCGCTGA CGCTGAGTAG GCTCCTATGC CGCTAGC...
Consensus  GTGCGGCACG AAGCCGCTGA CGCcGAGTcG GCTCCTATGC CGCTAGCtat

           51                                               100
       Cm  GCCCTGCTTG ....GTCCCC C......GGC ACGGTGGAGG TTCCGTAGCG
       Cr  GCCCTGCTTG ....GTCCCC C......GGC ACGGTGGAGG TTCCGTAGCG
      Cme  GCCCTGCTTG ....GTCCCC C......GGC ACGGTGGAGG TTCCGTAGCG
       Pt  GCCCTGCTTG ....GTCCCC C......GGC ACGGTGGAGG TTCCGTAGCG
       Sl  GCCCTGCTTG ....GTCCCC CTCCCCCGGC ACGGTGGAGG TTCCGTAGCA
     Pv-C  GCCCTGCTTG CTTGGTCCCC C......GGC ACGGTGGAGG TCCCGTAGCG
       Vc  GCCCTGCTTG ....GTCCCC C......GGC ACGGTGGAGG TTCCGTAGCG
       Cd  .......... .......... .......... ACGGTGGAGG TTCCGTAGCG
       Ca  .......... .......... .......... ACGGTGGAGG TTCCGTAGCG
       Cp  .......... .......... .......... ACGGTGGAGG TTCCGTAGCG
Consensus  gccctgcttg ....gtcccc c......ggc ACGGTGGAGG TtCCGTAGCG

           101                                              150
       Cm  CGTCATGAGC ACCGGGC..T AAGGGGCGGT TGAGCAACTC AAGCGAACCG
       Cr  CGTCATGAGC ACCGGGC..T AAGGGGCGGT TGAGCAACTC AAGCGAACCG
      Cme  CGTCATGAGC ACCGGGC... .......GGT TGAGCAACTC AAGCGAACCG
       Pt  CGTCATGAGC ACCGGGC..T AAGGGGCGGT TGAGCAACTC AAGCGAACCG
       Sl  CGTCATGAGC ACCGGGC..T AAGGGGCGGT TGAGCAACTC AAGCGAACCG
     Pv-C  CGTCATGAGC ACCGGGC..T AAGGGGCGGT TGAGCAACTC AAGCGAACCG
       Vc  CGTCATGAGC ACCGGGCGCT AAGGGGCGGT TGAGCAACTC AAGCGAACCG
       Cd  CGTCATGAGC ACCGGGC..A AAGGGACGGT TGAGCAACTC AAGCGAACCG
       Ca  CGTCATGAGC ACCGGGC..A AAGGGACGGT TGAGCAACTC AAGCGAACCG
       Cp  CGTCATGAGC ACCGGGC..A AAGGGACGGT TGAGCAACTC AAGCGAACCG
Consensus  CGTCATGAGC ACCGGGC..t AAGGGgCGGT TGAGCAACTC AAGCGAACCG

           151                                              200
       Cm  CCCTACCTTA CTACAACATA GGGACAGAAG GGAGAAGGTT GTGAAGGTGG
       Cr  CCCTACCTTA CTACAACATA GGGACAGAAG GGAGAAGGTT GTGAAGGTGG
      Cme  CCCTACCTTA CTACAACATA GGGACAGAAG GGAGAAGGTT GTGAAGGTGG
       Pt  CCCTACCTTA CTACAACATA GGGACAGAAG GGAGAAGGTT GTGAAGGTGG
       Sl  CCTTACCTTA TTCCAACATA GGGACAGAAG GGAGAAGGTT GTGAAGGTGG
     Pv-C  CCCTACCTTA CTACAACATA AGGACAGAAG GGAGAAGGTT GTGAAGGTGG
       Vc  CCCTGCCTTA CTACAACATA GGGACAGAAG GGAGAAGGTT GTGAAGGTGG
       Cd  CCCTACCTGA CTTTGG.ATA AAGATAGAAG GGAGAAGGTT GTGAAGGTGG
       Ca  CTCTACCTGA CTTTGG.ATA AAGATAGAAG GGAGAAGGTT GTGAAGGTGG
       Cp  CTCTACCTGA CTTTGG.ATA AAGATAGAAG GGAGAAGGTT GTGAAGGTGG
Consensus  CcCTaCCTtA CTacaacATA ggGAcAGAAG GGAGAAGGTT GTGAAGGTGG
```

	201				250
Cm	CCTCGTTATC	CACACCTCCG	GTCGGATGAA	TGGAGGACCG	ACCGACCCGG
Cr	CCTCGTTATC	CACACCTCCG	GTCGGATGAA	TGGAGGACCG	ACCGACCCGG
Cme	CCTCGTTATC	CACACCTCCG	GTCGGATGAA	TGGAGGACCG	ACCGACCCGG
Pt	CCTCGTTATC	CACACCTCCG	GTCGGATGAA	TGGAGGACCG	ACCGACCCGG
Sl	CCTCGTTATC	CATACCTCCG	GTCGGATGAA	TGGAGGACCG	ACCGACCCGG
Pv-C	CCTCGTTATC	CACACCTCCG	GTCAGATGAA	TGGAGGACCG	ACC....CGG
Vc	CCTCGTTATC	CACACCTCCG	GTCGGATGAA	TGGAGGACCG	ACCGACCCGG
Cd	CCTCGTTATC	CACACATCTG	GTC.......	.GGAGGACCG	ACC....TGG
Ca	CCTCGTTATC	CACACATCTG	GTC.......	.GGAGGACCG	ACC....TGG
Cp	CCTCGTTATC	CACACATCTG	GTC.......	.GGAGGACCG	ACC....TGG
Consensus	CCTCGTTATC	CACACcTCcG	GTCggatgaa	tGGAGGACCG	ACCgacccGG

	251				300
Cm	GTTTTCATGA	GCGTTGGCGG	GTCCTGGAGT	GCCTGTCAAG	GGCGCTAGCG
Cr	GTTTTCATGA	GCGTTGGCGG	GTCCTGGAGT	GCCTGTCAAG	GGCGCTAGCG
Cme	GTTTTCATGA	GCGTTGGCGG	GTCCTGGAGT	GCCTGTCAAG	GGCGCTAGCG
Pt	GTTTTCATGA	GCGTTGGCGG	GTCCTGGAGT	GCCTGTCAAG	GGCGCTAGCG
Sl	GTTTTCACGA	GCGTTGGCGG	GTTCTGGAGT	GCCTGTCAAG	GGCGCTAGCG
Pv-C	GTTTTCATGA	GCGTTGGCGG	GTCCTGGAGT	GCCTGTCAAG	GGCGCTAGCG
Vc	GTTTTCACGA	GCGTTGGCGG	GTTCTGGAGT	GCCTGTCAAG	GGCGCTAGCG
Cd	GTTTTCACGA	GCGTAGGCGG	GTCCTGGAGT	GCCCGTCAAG	GGCGCTAGCG
Ca	GTTTTCACGA	GCGTAGGCGG	GTCCTGGAGT	GCCCGTCAAG	GGCGCTAGCG
Cp	GTTTTCACGA	GCGTAGGCGG	GTCCTGGAGT	GCCCGTCAAG	GGCGCTAGCG
Consensus	GTTTTCAcGA	GCGTtGGCGG	GTcCTGGAGT	GCCtGTCAAG	GGCGCTAGCG

	301				350
Cm	CATACCCCGG	GGTGATCATC	ACCACCTGCA	CCTCACATCT	CGGCGCAGTG
Cr	CATACCCCGG	GGTGATCATC	ACCACCTGCA	CCTCACATCT	CGGCGCAGTG
Cme	CATACCCCGG	GGTGATCATC	ACCACCTGCA	CCTCACATCT	CGGCGCAGTG
Pt	CATACCCCGG	GGTGATCATC	ACCACCTGCA	CCTCACATCT	CGGCGCAGTG
Sl	CATACCCCGG	GGTGATCATC	ACCACCTGCA	CCTCACATCT	CGGCACAGTG
Pv-C	CATACCCCGG	GGTGATCATC	ACCACCTGCA	CCTCACATCT	CGGCACAGCG
Vc	CATACCCCGG	GGTGATCATC	ACCACCTGCA	CCTCACATCT	CGGCACAGTG
Cd	CATACCCCGG	GGTGATCATC	ACCACCTGCA	CCTCACATCT	CGGCACAGTG
Ca	CATACCCCGG	GGAGATCATC	ACCACCTGCA	CCTCACATCT	CGGCACAGTG
Cp	CATACCCCGG	GGAGATCATC	ACCACCTGCA	CCTCACATCT	CGGCACAGTG
Consensus	CATACCCCGG	GGtGATCATC	ACCACCTGCA	CCTCACATCT	CGGCaCAGtG

	351				400
Cm	GAACGTGTAA	CCCGCCTGCT	GTCTCATTCA	ACTACATTTG	TTCCTGTAAT
Cr	GAACGTGTAA	CCCGCCTGCT	GTCTCATTCA	ACTACATTTG	TTCCTGTAAT
Cme	GAACGTGTAA	CCCGCCTGCT	GTCTCATTCA	ACTACATTTG	TTCCTGTAAT
Pt	GAACGTGTAA	CCCGCCTGCT	GTCTCATTCA	ACTACATTTG	TTCCTGTAAT
Sl	GAACGTGTAA	CCCGCCTGCT	GTTCCATTCA	ACTACATTTG	TTCCTTTAAT
Pv-C	GAACGTGTAA	CCCGCCTGCT	GTCTCATTCA	ACTACATTTG	TTCCTGTAAT
Vc	GAACGTGTAA	CCCGCCTGCT	GTTTCATTCA	ACTACATTTG	TTCCTGTAAT
Cd	GAACGTGTAA	CCCGCCTGCT	GTCCAAGTCA	ACCACTGAAT	TTCCTGTAAT
Ca	GAACGTGTAA	CCCGCCTGCT	GTCCAAGTCA	ACCACTTAAT	TTCCTGTAAT
Cp	GAACGTGTAA	CCCGCCTGCT	GTCCAAGTCA	ACCACTTAAT	TTCCTGTAAT
Consensus	GAACGTGTAA	CCCGCCTGCT	GTctcAtTCA	ACtACatttg	TTCCTGTAAT

```
           401                                               450
       Cm  CTATAGC..C TAACAGAACG CAGCAGCGAG GGACAACCCG CCCATACAG.
       Cr  CTATAGC..C TAACAGAACG CAGCAGCGAG GGACAACCCG CCCATACAG.
       Cm  CTATAGC..C TAACAGAACG CAGCAGCGAG GGACAACCCG CCCATACAG.
      Pte  CTATAGC..C TAACAGAACG CAGCAGCGAG GGACAACCCG CCCATACAG.
       Sl  CCATAGC..T TAAAAGAACG CAGCAGCGAG GGACAACCCG CCCATACAG.
     Pv-C  CCATAGC..C TAACAGAACG CAGCAGCGAG GGACAACCCG CCCATACAG.
       Vc  CCATAGC..C TAACAAAACG CAGCAGCGAG GGACAACTCG CCCATACAG.
       Cd  TCATAGCGCC TAACAGAACG TAGCAGCAAG GGACAACCCA CCCATACAGA
       Ca  TCATAGCACC TAACAGAACG CAGCAGCAAG GGACAACCCA CCCATACAGA
       Cp  TCATAGCACC TAACAGAACG CAGCAGCAAG GGACAACCCA CCCATACAGA
Consensus  ccATAGC..C TAACAgAACG cAGCAGCgAG GGACAACcCg CCCATACAG.

           451                                               500
       Cm  ...CCAGCGG GGAGGATGGC ACTACTGGCA AAGACCGTCT GGCGAAAACG
       Cr  ...CCAGCGG GGAGGATGGC ACTACTGGCA AAGACCGTCT GGCGAAAACG
      Cme  ...CCAGCGG GGAGGATGGC ACTACTGGCA AAGACCGTCT GGCGAAAACG
       Pt  ...CCAGCGG GGAGGATGGC ACTACTGGCA AAGACCGTCT GGCGAAAACG
       Sl  ...CCAGCGG GGAGGATGGC ACTACTGGCA AAGACCGTCT GGCGAAAACG
     Pv-C  ...CCAGCGG GGAGGATGGC ACTACTGGCA AAGACCGTCC GGCGAAAACG
       Vc  ...CCAGCGG GGAGGATGGC ACTACTGGCA AAGACCGTCT GGCGAAAACG
       Cd  CAGCCTGCGG GGAGGATGGC ACTACTGGCA AAGACCGTCT GGCGAAAACG
       Ca  CAGCCTGCGG GGAGGATGGC ACTACTGGCA AAGACCGTCT GGCGAAAACG
       Cp  CAGCCTGCGG GGAGGATGGC ACTACTGGCA AAGACCGTCT GGCGAAAACG
Consensus  ...CCaGCGG GGAGGATGGC ACTACTGGCA AAGACCGTCt GGCGAAAACG

           501                                               550
       Cm  CCGCAGGCGC GAAGCGTGGT AGGCCTGCGC CGGGGGAGCA TAGCATAGGG
       Cr  CCGCAGGCGC GAAGCGTGGT AGGCCTGCGC CGGGGGAGCA TAGCATAGGG
      Cme  CCGCAGGCGC GAAGCGTGGT AGGCCTGCGC CGGGGGAGCA TAGCATAGGG
       Pt  CCGCAGGCGC GAAGCGTGGT AGGCCTGCGC CGGGGGAGCA TAGCATAGGG
       Sl  CCGCAGGCGC GAAGCGTGGT AGGCCTGCGC CGGGGGAGCA TACCATAGGG
     Pv-C  CCGCAGGCGC GAAGCGTGGT AGGCCTGTGC CGGGGGAGCA TAGCATAGGT
       Vc  CCGCAGGCGC GAAGCGTGGT AGGCCTGCGC CGGGGGAGCA TAGCATAGGG
       Cd  CCGCAGGCGC GAAGCGTGGT GGGCCTGCGC CGGGGGAGCA TA.....GGG
       Ca  CCGCTGGCGC GAAGCGTGGT GGGCCTGCGC CGGGGGAGCA TA.....GGG
       Cp  CCGCTGGCGC GAAGCGTGGT GGGCCTGCGC CGGGGGAGCA TA.....GGG
Consensus  CCGCaGGCGC GAAGCGTGGT aGGCCTGcGC CGGGGGAGCA TAgcataGGg

           551                                               600
       Cm  AGGAAAGGGA GCCCGGACGG TGGAAGAGCC AGGGGAGGCC GGGTCATTTA
       Cr  AGGAAAGGGA GCCCGGACGG TGGAAGAGCC AGGGGAGGCC GGGTCATTTA
      Cme  AGGAAAGGGA GCCCGGACGG TGGAAGAGCC AGGGGAGGCC GGGTCATTTA
       Pt  AGGAAAGG.. .......CGG TGGAAGAGCC AGGGGAGGCC GGGTCATTTA
       Sl  AGGAAAGGGA GCCCGGACGG TGGAAGAGCC AGGGGAGGCC GGGTCATTTG
     Pv-C  AGGAAAGGGA GCCAGGACGG TGGAAGAGCC AGGGGGGGCC GGGTCATTTG
       Vc  AGGAAAGGGA GCCCGGACGG TGGAAGAGCC AGGGGAGGCC GGGTCATTTG
       Cd  AGGAAAGGGA GCCCGGACGG TGGAAGAGCC AGGGGAAGCC GGGTCATTTG
       Ca  AGGAAAGGGA GCCCGGACGG TGGAAGAGCC AGGGGAAGCC GGGTCATTTG
       Cp  AGGAAAGGGA GCCCGGACGG TGGAAGAGCC AGGGGAAGCC GGGTCATTTG
Consensus  AGGAAAGGGA GCCcGGACGG TGGAAGAGCC AGGGGagGCC GGGTCATTTg
```

```
              601                                                  650
          Cm  ACGGAAATGG AAGG.CTTTT CCCCTATTAT AGTATAGAAG GCCCTATGAA
          Cr  ACGGAAATGG AAGG.CTTTT CCC....... .GTATAGAAG GCCCTATGAA
         Cme  ACGGAAATGG AAGG.CTTTT CCCCTATTAT AGTATAGAAG GCCCTATGAA
          Pt  ACGGAAATGG AAGG.CTTTT CCC....... .GTATAGAAG GCCCTATGAA
          Sl  ACGGAAATGG AAGG.CTTTT CCC......T ATTAGAGAAG GCCCTATGGA
        Pv-C  ACGGAAATGG AAGG.CTTTT CCCC.....T ATTAGAGAAG GCCCTCTGAA
          Vc  ACGGAAATGG AAGG.ATTTT A......... ...AAAGAAA .....ATGAA
          Cd  ACGGAAATGG AAGG...... CGGCTCATTC ATTCTTGAAA AAAGAGGGGG
          Ca  ACGGAAATGG AAGGTCCGAG CAGCTCATTC ATTCTTGGAA AAAGAGGGGG
          Cp  ACGGAAATGG AAGGTCCGAG CAGCTCATTC ATTCTTGGAA AAAGAGGGGG
   Consensus  ACGGAAATGG AAGG.ctttt c..c...... attataGaAa .....atGaa

              651                                                  700
          Cm  .......... AGTGCATGAA TTCTTGGAAA AAGAGGGAGC GAGCCTATAT
          Cr  .......... AGTGCATGAA TTCTTGGAAA AAGAGGGAGC GAGCCTATAT
         Cme  .......... AGTGCATGAA TTCTTGGAAA AAGAGGGAGC GAGCCTATAT
          Pt  .......... AGTGCATGAA TTCTTGG... ......GAGC GAGCCTATAT
          Sl  GT.AAGGGGA AGTGGATGAA TTCTTGGAAA AAGAGGGAGC GAGCCTATAT
        Pv-C  GTCAAGGGGA AGTGCATTCA TTCTTGGAAA AAGAGGGAGC AAGCCTATAA
          Vc  .......... .........A TTC....... .......... ..........
          Cd  GGAGCGAGCC AATGTATCAA TGAATAGATA CAGTCAACGG TAGACAGACA
          Ca  GGAGCGAGCC AATGTATCAA TGAATAGATA CAGTCAACGG TAGACAGACA
          Cp  GGAGCGAGCC AATGTATCAA TGAATAGATA CAGTCAACGG TAGACAGACA
   Consensus  g....g.g.. a.tg.at.aA Ttc.t.ga.a .ag.....g. .ag.c.....

              701                                                  750
          Cm  ...CAAA... ..AATGTAAT ACAGTAAATT CAATGAATAG ATAGAGTCAA
          Cr  ...CAAA... ..AATGTAAT AAAGTAAATT CAATGAATAG ATAGAGTCAA
         Cme  ...CAAA... ..AATGTAAT AAAGTAAATT CAATGAATAG ATAGAGTCAA
          Pt  ...CAAA... ..AATGTAAT AAAGTAAATT CAATGAATAG ATAGAGTCAA
          Sl  ...AAAATAG AAAATGAAAT AAAGAAAATT CCATGAATAG ATAGAGTCAA
        Pv-C  ...AAA.... ....TGAATG AAAG...... ...TGAATAG ATAGAGTCAA
          Vc  .......... .......... .......... .......... ATAGGATAAA
          Cd  GCGCTGCCTA CACGCGAATT TGCTTCCGAA CAAGCAACGG ATTGAGCAAC
          Ca  GCGCTGCCTA CACGCGAATT AGCTTCCGAA CAAGCAAGGG ATTGAGCAAC
          Cp  GCGCTGCCTA CACGCGAATT AGCTTCCGAA CAAGCAAGGG ATTGAGCAAC
   Consensus  ...c...... .....gaa.t a...t..... caa..aa..g ATaGagtaAa

              751                                                  800
          Cm  CGGTACGACA GACAGCGCTG CCTACACGCG AA..TTAGCT TCCGAGGTCG
          Cr  CGGTACGACA GACAGCGCTG CCTACACGCG AA..TTCGCT TCCGAGGTCG
         Cme  CGGTACGACA GACAGCGCTG CCTACACGCG AA..TTAGCT TCCGAGGTCG
          Pt  CGGTACGACA GACAGCGCTG CCTACACGCG AA..TTAGCT TCCGAGGTCG
          Sl  CGGTACGACA GACAGCGCTG CCTACACGCG AA..TTAGCT TCCGAGGTTG
        Pv-C  CGGTACGACA GACAGCGCTG CCTACACGCG AA..TTAGCT TCCGAGGTCG
          Vc  CGGTACGACA GACAGGGCTG CCGACACGCG AA..TTAGCT TCCGAGGTCG
          Cd  TAGCGCGAAA GCCGTTGC.G CTAACGCGCA TCCGTTTTCT TGC.TGGTCG
          Ca  TAGCGCGA.. ..CATTGC.G CTAACGCGCA TCCGTTTTCT TGC.TGGTCG
          Cp  TAGCGCGAAA GCCGTTGC.G CTAACGCGCA TCCGTTTTCT TGC.TGGTCG
   Consensus  cgGtaCGAca gaCag.GCtG Cc.ACaCGCg aa..TTagCT TcCgaGGTCG
```

```
           801                                                 850
       Cm  AGCGGTCTCA ATTTCACTAC AGGATTTGCG AATGAATGCT GGGCTGGACC
       Cr  AGCGGTCTCA ATTTCACTAC AGGATTTGCG AATGAATGCT GGGCTGGACC
      Cme  AGCGGTCTCA ATTTCACTAC AGGATTTGCG AATGAATGCT GGGCTGGACC
       Pt  AGCGGTCTCA ATTTCACTAC AGGATTTGCG AATGAATGCT GGGCTGGACC
       Sl  AGCAGTCTCA ATTTCACTAC AGGATTTGCG AATGAATGCT GGGCTGGGCC
     Pv-C  AGCAGTCTCA ATTTCACTAC AGGATTTGCG AATGAATGCT GGGCTGGGCC
       Vc  AGCAGTCTCA ATTTCACTAC AGGATTTTCG AATGAATGCT GGGCTGGGCC
       Cd  AGCAGTCTCA ATTTCACTAC AGGATTTGCG AATGAATGCT GGGCTGGGCC
       Ca  AGCAGTCTCA ATTTCACTAC AGGATTTGCG AATGAATGCT GGGCTGGGCC
       Cp  AGCAGTCTCA ATTTCACTAC AGGATTTGCG AATGAATGCT GGGCTGGGCC
Consensus  AGCaGTCTCA ATTTCACTAC AGGATTTgCG AATGAATGCT GGGCTGGgCC

           851                                                 900
       Cm  ACCTCGAATG GCGTGAGCCG CATGCGGGGA GACCCGCACG TACGGTTTTT
       Cr  ACCTCGAATG GCGTGAGCCG CATGCGGGGA GACCCGCACG TACGGTTTTT
      Cme  GCCTCGAATG GCGTGAGCCG CATGCGGGGA GACCCGCACG TACGGTTTTT
       Pt  ACCTCGAATG GCGTGAGCCG CATGCGGGGA GACCCGCACG TACGGTTTTT
       Sl  ACCTCGAATG GCGTGAGCCG CATGCGGGGA GACCCGCACG TACGGTTTTT
     Pv-C  ACCTCGAATG GCGTGAGCCG CATGCGGGGA GACCCGCACG TACGGTTTTT
       Vc  ACCTCGAATG GCGTGAGCCG CATGCGGGGA GACCCGCACG TACGGTTTTT
       Cd  ACCTCAAATG GCGTGAGCCG CATGCGGGGA GACCCGCACG TACGGTTTTC
       Ca  ACCTCAAATG GCGTGAGCCG CATGCGGGGA GACCCGCACG TACGGTTTTC
       Cp  ACCTCAAATG GCGTGAGCCG CATGCGGGGA GACCCGCACG TACGGTTTTC
Consensus  ACCTCgAATG GCGTGAGCCG CATGCGGGGA GACCCGCACG TACGGTTTTt

           901                                      940
       Cm  AGGGGGATCT GGTCGAAAGA CCGGCCGGCG CCCACCCGAC
       Cr  AGGGGGATCT GGTCGAAAGA CCGGCCGGCG CCCACCCGAC
      Cme  AGGGGGATCT GGTCAAAAGA CCGGCCGGCG CCCACCCGAC
       Pt  AGGGGGATCT GGTCGAAAGA CCGGCCGGCG CCCACCCGAC
       Sl  AGGGGGATCT GGTCGAAAAA CCGGCCGGCG CCCACCCGAC
     Pv-C  AGGGGGATCT GGTCGAAAGA CCGGCCGGCG CCCACCCGAC
       Vc  AGGGGGATCT GGTCGAAAGA CCGGCCGGCG CCCACCCGAC
       Cd  AGGGGGATCC GG........ CCGGCCGGCG CCCACCCGAC
       Ca  AGGGGGATCC GG........ CTGGCCGGCG CCCACCCGAC
       Cp  AGGGGGATCC GG........ CTGGCCGGCG CCCACCCGAC
Consensus  AGGGGGATCt GGtcgaaaga CcGGCCGGCG CCCACCCGAC
```

Figure S3. *nad7*i1 sequence alignments. DNA sequences of *nad7*i1 PCR amplification products were aligned on the Multalin (Corpet, 1988) interface web server <http://multalin.toulouse.inra.fr/multalin/> accessed 8/26/2022. The *nad7*i1 sequences of *Citrus maxima* (*Cm*), *Citrus reticulata* (*Cr*), *Citrus medica* (*Cme*), *Poncirus trifoliata* (*Pt*), *Solanum lycopersicum* (*Sl*), *Phaseolus vulgaris* 'Calima' (*Pv*-C), *Vaccinium corymbosum* (*Vc*), *Cynodon dactylon* (*Cd*), *Cenchrus americanus* (*Ca*), and *Cenchrus purpureus* (*Cp*) were aligned revealing multiple indel and SNP polymorphisms between genera. *Citrus* species were distinguished by indels at positions 120-127, 559-567, 624-631 and 678-686 in the alignment. The *Cenchrus* species were distinguished by a four nucleotide indel position 759-762 and a SNP at position 764 in the multi-species alignment. *Citrus* congener species *C. paradisi*, and *C. japonica* did not differ from *Cm* and were not included in the alignment. This was also the case for *Solanum pennellii* as compared to *Sl*, *Vaccinium virgatum* as compared to *Vc*, *Cynodon transvaalensis* as compared to *Cd* and for *Phaseolus vulgaris* 'Jamapa' as compared to *Pv*-C.
SNP and indel polymorphisms that distinguish congener species are highlighted in yellow.
